# *FRMPD4*, a causal gene for intellectual disability and epilepsy, is associated with X-linked non-syndromic hearing loss

**DOI:** 10.64898/2026.03.27.26349271

**Published:** 2026-03-30

**Authors:** Daniel Liedtke, Kristen Rak, Katrina M Schrode, Philip Hehlert, Niloofar Chamanrou, Daniel Bengl, Radoslaw Katana, Soganad Heydaran, Julia Doll, Mei Han, Indrajit Nanda, Pingkalai R Senthilan, Lukas Jürgens, Linda Bieniussa, Johannes Voelker, Cordula Neuner, Michaela AH Hofrichter, Jörg Schröder, Renske T.W. Schellens, Erik de Vrieze, Erwin van Wijk, Ulrich Zechner, Stefan Herms, Per Hoffmann, Tobias Müller, Marcus Dittrich, Oliver Bartsch, Peter M Krawitz, Eva Klopocki, Wafaa Shehata-Dieler, Reza Maroofian, Tao Wang, Paul F Worley, Martin C Göpfert, Hamid Galehdari, Amanda M Lauer, Thomas Haaf, Barbara Vona

**Affiliations:** 1 Institute of Clinical Genetics and Genomic Medicine, University Hospital Würzburg, Würzburg, Germany; 2 Institute of Human Genetics, Julius-Maximilians-University Würzburg, Würzburg, Germany; 3 Department of Oto-Rhino-Laryngology, Head and Neck Surgery and the Comprehensive Hearing Center, University Hospital Würzburg, Würzburg, Germany; 4 Department of Otolaryngology, Johns Hopkins University School of Medicine, Baltimore, Maryland, USA; 5 Department of Cellular Neurobiology, University of Göttingen, Göttingen, Germany; 6 Department of Biology, Faculty of Science, Shahid Chamran University of Ahvaz, Ahvaz 6135783151, Iran; 7 Department of Genetic Medicine, Johns Hopkins University School of Medicine, Baltimore, Maryland, USA; 8 Neurobiology and Genetics, Theodor-Boveri-Institute, Biocenter, Julius-Maximilians-University of Würzburg, Am Hubland, Würzburg, Germany; 9 Department of Otorhinolaryngology, Radboud University Medical Center, 6525 GA Nijmegen, The Netherlands; 10 Institute of Human Genetics, University Medical Center, Johannes Gutenberg University, Mainz, Germany; 11 Institute of Human Genetics, University of Bonn, Bonn, Germany; 12 Institute of Medical Genetics and Pathology, University Hospital Basel, Switzerland; 13 Human Genomic Research Group, Department of Biomedicine, University of Basel, Switzerland; 14 Institute of Neuroscience and Medicine (INM-1), Research Center Jülich, Jülich, Germany; 15 Department of Bioinformatics, Biocenter, Julius-Maximilians-University Würzburg, Würzburg, Germany; 16 Human Genetics Unit, Medical Care Centre, Johannes Gutenberg University Mainz, Mainz, Germany; 17 Institute for Genomic Statistics and Bioinformatics, University of Bonn, Bonn, Germany; 18 Department of Neuromuscular Disorders, UCL Queen Square Institute of Neurology, University College London, London, WC1N 3BG, UK; 19 Department of Neuroscience, Johns Hopkins University School of Medicine, Baltimore, Maryland, USA; 20 Institute for Auditory Neuroscience and Inner Ear Lab, University Medical Center Göttingen, Göttingen, Germany; 21 Auditory Neuroscience and Optogenetics Laboratory, German Primate Center, Göttingen, Germany; 22 Collaborative Research Center 1690 (CRC1690), University of Göttingen, Göttingen, Germany; 23 Department of Obstetrics and Gynecology, Brigham and Women’s Hospital, Harvard Medical School, Boston, MA, USA; 24 Program in Medical and Population Genetics, Broad Institute of MIT and Harvard, Cambridge, MA, USA

**Keywords:** *Drosophila* model, FERM and PDZ domains containing protein 4 (*FRMPD4*), non-syndromic hearing loss, novel gene discovery, mouse model, sensorineural hearing loss, X-linked, zebrafish model

## Abstract

**Background:**

Understanding the phenotypic spectrum of disease-associated genes is essential for accurate diagnosis and targeted therapy. *FRMPD4* (FERM and PDZ Domain Containing 4) has previously been associated with intellectual disability and epilepsy. However, its potential role in non-syndromic hearing loss has not been explored.

**Methods:**

We performed genetic analysis in two unrelated families presenting with non-syndromic sensorineural hearing loss, identifying maternally inherited missense variants in *FRMPD4*. Clinical phenotyping included audiological assessment and evaluation for neurodevelopmental involvement. Cross-species expression analyses were conducted in *Drosophila*, zebrafish, and mouse. Functional characterization included quantitative evaluation of sound-evoked responses in *Drosophila nicht gut hörend* (*ngh*) mutants, assessment of neuronal development and acoustic startle responses in zebrafish loss of function models, and morphological cochlear analyses with auditory brainstem response measurements in knockout mice.

**Results:**

Three affected males from two unrelated families presented with prelingual, bilaterally symmetrical sensorineural hearing loss, with confirmed congenital onset in one individual and no evidence of neurodevelopmental abnormalities. Cross-species analyses demonstrated evolutionarily conserved expression of *FRMPD4* in auditory structures. In *Drosophila*, quantitative analysis of sound-evoked responses in *ngh* mutants revealed impaired auditory function. Zebrafish loss of function models exhibited reduced neuronal populations in the otic vesicle and posterior lateral line, abnormal neuromast development, and diminished acoustic startle responses. In mice, *Frmpd4* knockout resulted in high-frequency hearing loss and cochlear abnormalities consistent with the human phenotype.

**Conclusions:**

Our findings expand the phenotypic spectrum of *FRMPD4* to include non-syndromic sensorineural hearing loss and establish its evolutionarily conserved role in auditory function. These results have direct implications for genetic diagnosis and variant interpretation in patients with hearing loss.

## Background

Hereditary non-syndromic hearing loss is one of the most common and genetically heterogeneous sensory disorders. It affects approximately one to two per 1000 newborns, with genetic factors accounting for the majority of cases (Morton and Nance 2006). Autosomal recessive and dominant forms together account for roughly 95% of inherited non-syndromic hearing loss (Vona et al. 2020). Of the more than 155 genes currently associated with non-syndromic hearing loss, only five map to the X chromosome (*AIFM1, POU3F4*, *COL4A6*, *PRPS1*, and *SMPX*). Accordingly, X-linked forms are very rare and are estimated to account for 2–5% of non-syndromic hearing loss (Vona et al. 2020).

Genetic studies of non-syndromic hearing loss have uncovered numerous examples of pleiotropy, in which genes initially implicated in syndromic or neurodevelopmental disorders also contribute to isolated auditory phenotypes (Murray et al. 2017; Rehman et al. 2014; Riazuddin et al. 2009; Richard et al. 2021). These findings highlight shared molecular pathways between the auditory system and other organ systems and underscore the importance of reevaluating the phenotypic spectrum of disease-associated genes as new functions which are revealed through comprehensive genetic and functional studies (Tshering et al. 2025; Vona 2024).

The evolutionary conservation of many hearing-associated genes across vertebrate species further supports their essential role in auditory function. These genes frequently exhibit conserved expression in auditory organs, and functional studies have shown that their encoded proteins are essential for species-specific auditory function (Elkon et al. 2015; Pei et al. 2016). This suggests a common evolutionary origin for key auditory mechanisms and reveals conserved cellular functions (Carey and Amin 2006; Fritzsch and Straka 2014; Streit 2001). Beyond rodent models, other organisms such as zebrafish (*Danio rerio*) (Haddon and Lewis 1996; Raible and Kruse 2000) and *Drosophila melanogaster* (Li et al. 2018) have become powerful systems for studying gene function in hearing, despite species-specific adaptations in hearing organ structure and neuronal sound processing. For example, sound detection in zebrafish partly relies on neuromast hair cells of the anterior and posterior lateral line, which sense fluid movement along the body axis. These hair cells are homologs of the inner-ear hair cells and are evolutionarily related not only to hair cells in the human organ of Corti but also to the chordotonal mechanosensory neurons mediating hearing in *Drosophila* (Fritzsch and Straka 2014; Hassan and Bellen 2000; Nicolson 2017; Nicolson 2005).

In this study, we identify two novel missense variants in *FRMPD4* (OMIM: 300838) through exome sequencing and analysis of two previously undiagnosed families with non-syndromic hearing loss. *FRMPD4* has been linked to schizophrenia and intellectual disability (Hu et al. 2016; Matosin et al. 2016; Trujillano et al. 2017) and plays a functional role in dendritic outgrowth and morphogenesis (Lee et al. 2008). It has also been shown to interact with whirlin as part of the Usher syndrome type 2 protein complex in photoreceptor cells (Schellens et al. 2022), and *FRMPD4* pathogenic variants have been associated with isolated epilepsy and epilepsy with intellectual disability (Li et al. 2024). Here, we demonstrate that FRMPD4 additionally plays a conserved role in auditory pathways across humans, *Drosophila*, zebrafish, and mice, expanding its functional repertoire and implicating it in non-syndromic hearing loss.

## Methods

### Patient recruitment and clinical assessment

Families were recruited through a large rare disease study and through data sharing with collaborating clinicians. The sole inclusion criterion was the presence of hereditary hearing impairment. This study was approved by the Medical Faculty at the University of Würzburg (approval number: 46/15). Written informed consent was obtained from all participating individuals or their parental guardians prior to enrollment.

Otolaryngologic, audiological, and general medical data were ascertained from the medical records of both families and are described in detail in the Supplemental Materials and Methods.

### Molecular genetic work-up, exome sequencing, and data analysis

Genomic DNA (gDNA) was extracted from whole blood of affected individuals and available family members using a standard salt extraction method (Family 1: II:2, II:3, III:2, and III:3; Family 2: III:1, III:2, III:3, and IV:1). For Family 1, genome-wide SNP genotyping was performed using the Illumina Omni1-Quad SNP-array (Illumina, San Diego, CA, USA) according to the manufacturer’s specifications and analyzed as previously described (Vona et al. 2014). Subsequently, the gDNA of both affected children from Family 1 underwent targeted sequencing of an 80-gene deafness panel including genes associated with both syndromic and non-syndromic hearing loss. Sequencing was performed on an Illumina HiSeq2000 platform by Otogenetics Corporation (Norcross, GA, USA) and data were analyzed as described previously (Vona et al. 2014). Exome sequencing was performed on gDNA from the two affected boys and their parents from Family 1 (II:2, II:3, III:2, and III:3) using the SeqCap EZ Human Exome Library v3 (64M) enrichment kit (Roche NimbleGen). Libraries were sequenced as 2 × 100 bp paired-end reads on an Illumina HiSeq 2000 platform (Life and Brain GmbH, Bonn, Germany). For family 2, an exome library from the proband (IV:1) was prepared using the Agilent SureSelect v6 kit (Agilent, Santa Clara, CA, USA) and exome sequenced on an Illumina HiSeq4000 platform.

Exome data from Family 1 were aligned to the human reference genome (GRCh37/hg19) using the Cologne Center for Genomics Varbank v2.1 pipeline. This pipeline incorporates GATK for base recalibration, local alignment, and variant score recalibration. Variant calling was performed using MPILEUP, GATK and DINDEL according to best practice recommendations (McKenna et al. 2010). GeneTalk was additionally used for variant filtering and result validation (Kamphans and Krawitz 2012). Exome data from individuals in Family 2 was demultiplexed and aligned to the human reference genome (GRCh38) using Burrows-Wheeler Aligner for subsequent variant calling. Detailed exome filtering strategies, analysis workflows, and variant prioritization are described in the Supplemental Materials and Methods.

### Animal maintenance and experimentation

#### Drosophila

Fly stocks were maintained at 25°C on a standard cornmeal-agar diet. The mutants were obtained from the Bloomington Stock Center (BL 60756; genotype: *y*^*1*^ w^*^; Mi{MIC}CG42788^*MI02203*^). Reporter knockout (KO) line CG42788^1,Gal4,3xP3>DsRed^ (generated in this study) was used to investigate expression in Johnston’s organ in the second antennal segment.

Methods related to RT-PCR, immunofluorescence staining, and electrophysiological and mechanical recordings in *Drosophila* are included in the Supplemental Materials and Methods.

### Zebrafish

Zebrafish (*Danio rerio*) were bred and maintained in the aquatic facilities of the Biocenter of the Julius-Maximilians-University Würzburg, Germany according to FELASA guidelines (Aleström et al. 2020) (husbandry permit number 568/300–1870/13). Adult fish were kept at a mean temperature of 24–26°C in 10 l glass and 2.5 l plastic tanks, while embryos younger than 120 h post-fertilization (hpf) were raised at a temperature of 28.5°C in an incubator. A daily light cycle of 10 h dark/14 h light was maintained for breeding fish. Preconditioned reverse osmosis water with adjusted conductivity 500–1,100 μS/cm, pH 7.0 and stable water hardness was used. A food combination of *Artemia nauplii* and GEMMA Micro Food (age dependent sizes; Skretting, USA) was standard. All experimental procedures were performed according to the guidelines of the German animal welfare law and approved by the local government (Government of Lower Franconia; Tierschutzgesetz §11, Abs. 1, Nr. 1; Genotyping and startle response protocol permit number: DMS-2532-2-9 and DMS-2532-2-428). Zebrafish embryos (*Danio rerio*) of the *AB/TU* (ZDB-GENO-010924-10) and *AB/AB* (ZDB-GENO-960809-7) strains were used in this study and were staged by morphological characteristics (Kimmel et al. 1995). *frmpd4*^*sa12377*^ mutants were obtained from the European Zebrafish Resource Center (KIT, Karlsruhe, Germany; allele name: sa12377; ZFIN line ID: ZDB-ALT-130411-2117) and possess a G>T variant at an essential splice site in exon 11.

Methods related to whole mount *in situ* hybridization, Morpholino generation, CRISPR/Cas9 gene editing, RNA rescue via overexpression, DASPEI staining, immunofluorescence, scanning electron microscopy, and startle response testing with analysis are included in the Supplemental Materials and Methods.

### Mouse

Mouse breeding and procedures were conducted in strict accordance with NIH Guide for Care and Use of Laboratory Animals. Mouse studies were carried out in accordance with the Guide for the Care and Use of Laboratory Animals, the ARRIVE guidelines (Percie du Sert et al. 2020) and Johns Hopkins University Animal Care and Use Committee. Conditional KO *Frmpd4*^−/−^ mice (this mouse model was originally called *Preso1*^−/−^, hereafter called *Frmpd4*^−/−^ for simplicity) were generated as previously described and were viable, fertile, and showed similar development and breeding behaviors as wild type (WT) littermates (Hu et al. 2012). Briefly, exon 3 was deleted by inserting flanking *loxP* sites and a *loxP*/PGK-neo cassette and crossing with *CMV-cre* mice. The transgenic colony was maintained on a C57BL/6J background. Genotyping was performed as previously described using PCR of tail DNA (Hu et al. 2012). Mice were housed in temperature-controlled rooms with 12 h light/dark cycle and had free access to food and water.

Mouse experimental methods related expression analysis of *Frmpd4* in the cochlea and other tissues, auditory brainstem response (ABR) testing and analysis are described in Supplemental Materials and Methods. Primer sequences are listed in Supplementary Table 1.

### Statistical analysis

Relative distances of posterior lateral line primordium (PLL) migration, neuromast numbers in the cranial lateral line, and startle response reaction times were analyzed using OriginPro 2021 (OriginLab Corporation, Northampton, MA, USA) and visualized using boxplot/data point diagrams. Diagrams show whiskers indicating standard deviation (coefficient value: 1.5), the median (parallel line), the mean value (small box), and the upper and lower quartile (large box). For statistical analysis, the obtained data values were first tested for normal distribution (ANOVA test), and the significance was determined using a two-tailed Mann-Whitney-U test. An asterisk indicates significant changes between groups U < 0.01, while n.s. marks not significantly different groups. Generalized linear mixed model analysis was performed using the nlme package in R (Pinheiro et al. 2022; Vazquez et al. 2010). The model included frequency and genotype as factors and their interaction, as well as a random effect to account for repeated measures. Post hoc Tukey tests were computed to evaluate differences between genotypes by frequency. A *p*-value < 0.05 was considered statistically significant.

## Results

### Clinical evaluation of Family 1

**Figure 1.**
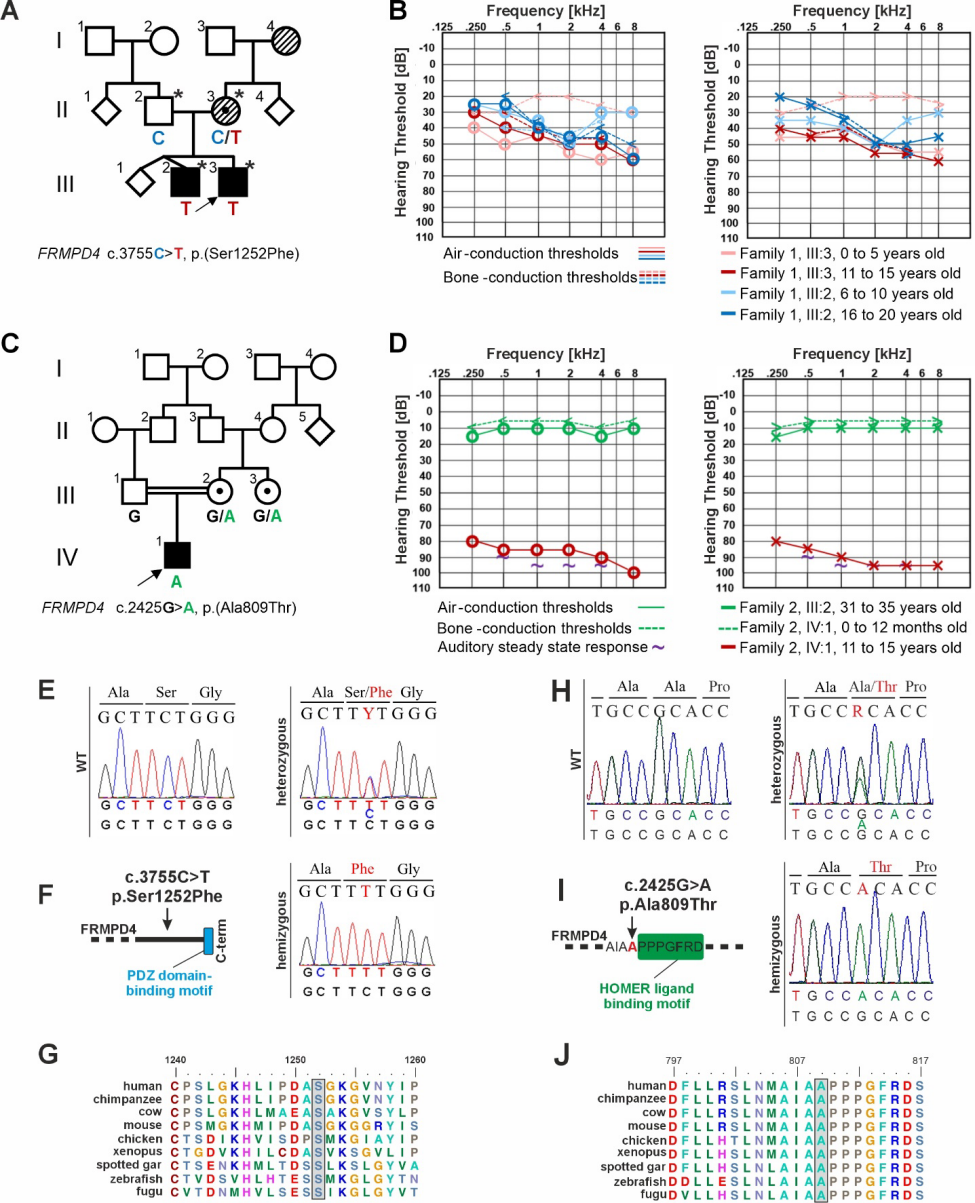
Identification of the putative disease-causing *FRMPD4* variant in two families with non-syndromic hearing loss. **(A)** Pedigree of family 1. Black symbols are affected individuals, unfilled symbols are unaffected individuals and individuals with stripes have late-onset hearing loss. Asterisks denote the family members whose exomes were sequenced. Genotypes at the c.3755C>T, p.(Ser1252Phe) position are denoted below each pedigree symbol. **(B)** Pure-tone audiograms of the affected males. Unmasked bone conduction measurements are included when available. Audiograms from III:3 at the age of 0–5 years (pink) and 11–15 years (red). Audiograms from III:2 at age 6–10 years (light blue) and 16–20 years (dark blue). Air- and bone-conduction thresholds are represented with circles and <, as well as crosses and > for right and left ears, respectively. **(C)** Pedigree of family 2 with genotypes of the *FRMPD4* c.2425G>A, p.(Ala809Thr) variant below each individual in whom segregation analysis was performed. **(D)** Audiometry from the proband (IV:1) included auditory steady-state responses at the age of 0–12 months (purple tilde), as well as a most recent pure-tone audiogram at the age of 11–15 years is in red. Pure-tone audiometry of the mother of the proband (III:2) at the age of 31–35 years is in green. **(E)** Direct sequencing confirmed the *FRMPD4* c.3755C>T, p.(Ser1252Phe) variant in family 1. The unaffected father was wild type (WT), the mother with self-reported mild hearing loss was heterozygous, and the two affected children were hemizygous. **(F)** The c.3755C>T, p.(Ser1252Phe) variant impacts an amino acid residue before the PDZ domain-binding motif. **(G)** Amino acid alignment of the p.Ser1252 region shows high evolutionary conservation in vertebrates. **(H)** Direct sequencing confirmed the *FRMPD4* c.2425G>A, p.(Ala809Thr) variant in family 2 with the mother of the proband (III:2) and maternal aunt (III:3) as heterozygous, the father of the proband (III:1) showing the WT allele, and the male proband with a hemizygous allele for the *FRMPD4* c.2425G>A, p.(Ala809Thr) variant. **(I)** The c.2425G>A, p.(Ala809Thr) variant impacts an amino acid residue directly before the HOMER ligand binding motif. **(J)** Amino acid alignment of the p.Ala809 region shows high evolutionary conservation in vertebrates.

Family 1 consists of a three-generation non-consanguineous family from Europe (Figure 1A). The youngest generation comprises three children, two of whom are affected by hearing loss. The youngest child (III:3) was born after an uneventful pregnancy. Following a failed newborn hearing screening, the child was diagnosed with mild-to-moderate sensorineural hearing loss shortly thereafter. The hearing of his older siblings was retrospectively evaluated, identifying mild sensorineural hearing loss in III:2. The older siblings were born after a complicated pregnancy after 35 weeks of gestation. Each had a birth weight of 2,120 g (19th percentile, Z-score: −0.88) and a body length of 46 cm (34th percentile, Z-score: −0.40). Individual III:1 was diagnosed with delayed speech and language development and received speech therapy. Otoscopy performed at the age of 6–10 years revealed normal closed tympanic membranes. Pure-tone audiometry and bilateral otoacoustic emissions were normal and reproducible. The most recent hearing test at 6–10 years of age confirmed normal hearing (data not shown), further arguing against a mitochondrial mode of inheritance.

Annual or semi-annual otoscopy and audiometric follow-up have been performed in both affected children. Bilateral distortion product and transient evoked otoacoustic emissions showed no reproducible responses in these individuals. Individual III:2 required early childhood speech therapy related to a delayed diagnosis. At most recent evaluation, hearing loss was moderate in individual III:3 (PTA_0.5–4kHz_ [pure-tone average] 46.25 dB HL [hearing level] [right] and PTA_0.5–4kHz_ 50 dB HL [left]), and mild in individual III:2 (PTA_0.5–4kHz_ 38.75 dB HL [right] and PTA_0.5–4kHz_ 40 dB HL [left]) at the age of 10–20 years, respectively (Figure 1B). Hearing loss was bilaterally symmetrical and stable over time. Speech audiometry without hearing aids performed in III:3 disclosed a 30% and 40% discrimination for left and right ears, respectively, that improved to 80% and 90% with hearing aids. Free-field audiometry without hearing aids revealed responses at stimulus levels between 60 and 70 dB. Hearing aids effectively rehabilitated hearing loss in both individuals. There has been no dizziness, ear pain, or ear discharge. Both attended mainstream schools, demonstrated good academic performance, and showed no concentration difficulties. Their school histories do not support developmental delay and intellectual disability. Pediatric developmental milestones followed an age-appropriate course. Whole blood analytics and electrocardiogram results were unremarkable. Clinical examination confirmed the absence of additional phenotypic abnormalities.

The mother (II:3) reported noticing mild hearing loss in her fourth decade of life, and the maternal grandmother (I:4) required hearing aids at age 55–60 years. Audiograms from both individuals are unavailable.

### Clinical evaluation of Family 2

Family 2 consists of a four-generation pedigree of Middle Eastern (Figure 1C). The proband (IV:1) was reported to have congenital bilateral non-syndromic hearing loss. Auditory steady-state response testing at 0–12 months of age estimated hearing thresholds of 90 dB bilaterally with an 85% confidence level (Figure 1D). Stimuli consisted of 100% amplitude-modulation and 20% frequency-modulated tones between 80 to 100 dB HL in 5 dB interval steps using headphones. Thresholds were estimated at 0.5, 1, 2, and 4 kHz in both ears. Otoacoustic emissions were absent. The child received cochlear implants in the prelingual stage. Pure tone audiometry at the age of 11–15 years confirmed severe sensorineural hearing loss (PTA_0.5–4kHz_ 86.25 dB HL) (Figure 1D). Tympanometric evaluation showed normal middle ear function. The proband’s mother (III:2) was confirmed to have normal hearing at the age of 30–35 years (Figure 1D), with speech recognition thresholds at 10 dB HL bilaterally and speech discrimination scores of 96% (right) and 92% (left).

### Exome sequencing identifies *FRMPD4* variants in patients with non-syndromic hearing loss

#### Family 1:

Genetic investigation was initiated following routine molecular diagnostic evaluation that failed to yield an informative diagnosis. This included analysis of *GJB2* and *STRC* with multiplex ligation-dependent probe amplification diagnostic testing of *GJB2*, *GJB3*, *GJB6*, *POU3F4* and *WFS1* (MRC-Holland). SNP-array analysis of the two affected individuals yielded an uninformative result (Illumina Omni1-Quad). An 80-gene deafness panel was subsequently performed but did not establish a genetic diagnosis.

gDNA from the parents and two affected children (II:2, II:3, III:2, and III:3) was subjected to exome sequencing (Figure 1A). Variant filtering disclosed 304,240 variants that were systematically filtered under autosomal recessive, autosomal dominant, and X-linked inheritance patterns (Supplementary Table 2). Fourteen heterozygous autosomal variants inherited from an unaffected parent were excluded (Supplementary Table 3). Analysis of variants in hearing loss-associated genes uncovered a heterozygous variant in *USH1C* (NM_153676.3:c.1591C>T, p.Arg531Cys), a gene causal for autosomal recessive Usher syndrome type 1 with congenital hearing impairment. This variant was classified as a variant of uncertain significance (VUS; PM2_P) and was excluded due to absence of a second variant and lack of clinical concordance (Supplementary Table 4). A likely benign (PM2_P, BP4_S) heterozygous *BDP1* (NM_018429.2:c.2351A>G, p.Lys784Arg) variant was also identified. Further exome-wide analysis uncovered a likely benign (PP3_P, BS2_S) hemizygous variant in *PHKA2* (NM_000292.2:c.202G>A, p.Asp68Asn) that was transmitted from the mother to both affected children. A heterozygous VUS (PM2_P, PP3_P) in *MYBPH* (NM_004997.2:c.989T>G, p.Leu330Arg) was identified. This gene has so far been characterized as a modifier of hypertrophic cardiomyopathy (Mouton et al. 2016) and with a putative skeletal muscle function (Mead et al. 2024) but no known association with hearing loss. A single missense variant in *FRMPD4* NM_001368397.1:c.3755C>T (p.Ser1252Phe) was identified in a hemizygous state in the two affected boys and was heterozygous in their mother that was confirmed with Sanger sequencing (Figure 1E). This variant is absent in gnomAD (n = 730,947 exomes and 76,215 genomes), TopMed (n = 138,000 genomes), the All of Us (n = 414,840 genomes), and the Varbank in house exome database (n = 511 exomes). *In silico* variant effect predictors suggested a nearly unanimous deleterious effect (Table 1). The cytosine at position c.3755 is highly conserved (phyloP: 4.64 [−14.1 to 6.4]) and occurs before the PDZ domain binding motif (Figure 1F). Similarly, the serine-to-phenylalanine substitution alters a highly conserved amino acid residue (Grantham distance: 155 [0–215]), with conservation observed in 48 of 75 species examined, including zebrafish (*Danio rerio*) and mouse (Figure 1G).

#### Family 2:

Exome sequencing of the proband of Family 2 (IV:1) uncovered 98,819 variants that were filtered according to all possible inheritance patterns (Supplementary Table 5). Analysis of hearing loss-associated genes uncovered a heterozygous *CDH23* (NM_022124.5:c.9014C>T, p.Ala3005Val) variant that was classified as VUS (PM2_P). Exome-wide analysis identified three homozygous variants in *SYPL2* (NM_001040709.1:c.406_407dup, p.Leu137Thrfs*21), *MAPKAPK5* (NM_139078.2:c.1099G>A, p.Gly367Ser), and *TNRC6C* (NM_001142640.2:c.2483G>T, p.Gly828Val) in genes that are not expressed in the inner ear, or associated with other phenotypes (Supplementary Table 6). Two further homozygous variants were identified in uncharacterized genes that were excluded from consideration on the basis of weak variant effect prediction scores (Supplementary Table 6). A single variant in *FRMPD4* (NM_014728.3:c.2425G>A, p.Ala809Thr) emerged as a candidate variant (Table 1, Supplementary Table 4). Presence of two independent families with missense variants in *FRMPD4* prompted further expression and functional analysis to associate *FRMPD4* with non-syndromic hearing loss.

### Comparison of the *FRMPD4* variants associated with non-syndromic hearing loss with variants causing intellectual disability

*FRMPD4* has a Residual Variation Intolerance Score of −1.84 ranking *FRMPD4* in the top 2% of genes most intolerant to variation. So far, 13 variants in *FRMPD4* have been associated with intellectual disability of varying severity, isolated epilepsy, or epilepsy with intellectual disability, the vast majority of which are maternally inherited (Supplementary Table 7), and so far, there is no clear distinct genotype-phenotype association. The two novel variants we describe that are associated with non-syndromic hearing loss do not appear to cluster to any one region of the gene or protein domain. Similarly, the previously described variants also appear to impact positions along the entire protein. A schematic overview of variant localization in the FRMPD4 protein is presented in Supplementary Figure 1.

### FRMPD4 orthologues are expressed in hearing organs during development

The high conservation of FRMPD4 variants identified in patients implied a potential function on hearing organs in other vertebrates. Genomic comparison between FRMPD4 orthologues showed relatively high amino acid conservation in the two functional domains, the PDZ and the FERM domain (Supplementary Figures 2A and 3). While mice share up to 99.3% amino acid-identity to the human protein domains, the zebrafish (up to 89.7% amino acid-identity) and even the *Drosophila* (up to 58.9% amino acid-identity) orthologues show remarkable conservation values. Synteny analyses further indicated the prolonged evolutionary conservation of the *frmpd4* locus in the investigated species (Supplementary Figure 2B).

**Figure 2.**
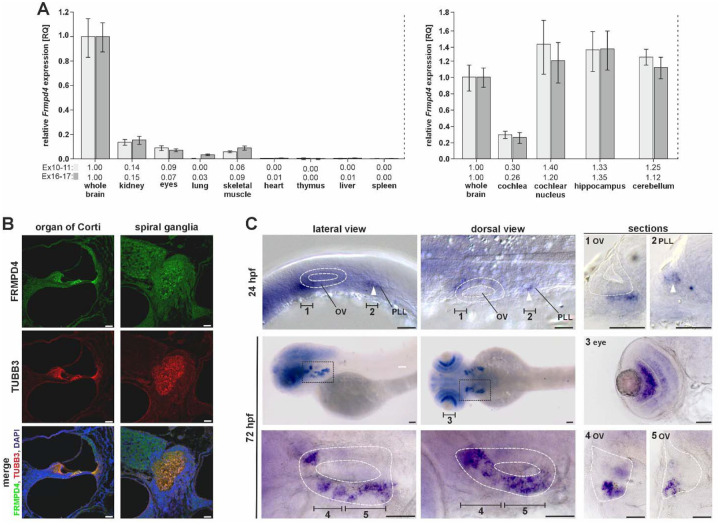
Frmpd4 expression in mice and zebrafish **(A)** Expression of *Frmpd4* in adult mice was detected mainly in whole brain, eyes, kidney, skeletal muscle, and in hearing organ tissues via qPCR. Expression in specific neuronal regions associated with hearing imply a relatively low *Frmpd4* expression in cochlear cells. **(B)** Localization of FRMPD4 and TUBB3 by immunofluorescence in cross-sections in the organ of Corti and in the spiral ganglion show very specific localization of FRMPD4 in distinct cell types, like phalangeal and hair cells (5 μm section; age: 3 mo). **(C)** Expression of *frmpd4* in zebrafish was investigated by *in situ* hybridization and was detected in the otic vesicle (OV; white dashed line) and in the posterior lateral line primordium (PLL; white arrowhead). Sectioning planes are indicated by black bars and their corresponding number (1: anterior otic vesicle 24 hpf; 2: lateral line primordium 24 hpf; 3: eye; 4: posterior otic vesicle 72 hpf; 5: anterior otic vesicle 72 hpf). Scale bars in B and C indicate 50 μm

To further test auditory-specific expression of FRMPD4 orthologues in vertebrates we investigated different animals for the presence of transcripts in their corresponding organs. *Frmpd4* transcripts in the mouse could be detected via qPCR in several neuronal tissues and kidney, but also in auditory organs, such as the cochlea and the cochlear nucleus (Figure 2A). *Frmpd4* was expressed nearly exclusively in the spiral ganglion neurons of the mouse at postnatal day 8 (P8). Expression in the spiral ganglion neurons increases between embryonic to early postnatal stages where it remains stably expressed between P8 and P30. Spiral ganglion neuron expression appears across types Ia, Ib, Ic and type 2 spiral ganglion neurons (Supplementary Figure 4). Validation of FRMPD4 localization by immunofluorescence on mouse cochlea supported the presence of FRMPD4 in organ of Corti, in the spiral ganglion and in the acoustic nerve during development (Figure 2B; Supplementary Figure 4).

Investigation of *frmpd4* expression in zebrafish was performed by whole-mount *in situ* hybridization to resolve spatio-temporal expression pattern changes during embryonic and early larval development (Figure 2C). *frmpd4* expression was detected in a broad, undefined expression domain in neuronal structures 24 hpf (hours post fertilization). Besides these domains, *frmpd4* transcripts were detected in cell clusters adjacent and anterior to the otic placode/otic vesicle (region 1 in Figure 2C) and in the lateral line primordium (region 2 in Figure 2C). Both regions are linked to the establishment of hearing organs in fish, as these precursor tissues will give rise to the inner ear and the lateral line organ during later stages of development (Haddon and Lewis 1996; Whitfield et al. 2002). In 72 hpf embryos, the expression of *frmpd4* is restricted to cells of the fore- and midbrain, the eyes and the otic vesicle, while expression in the lateral line was no longer detected at this time point. *frmpd4* expression in the otic placode/otic vesicle is restricted to distinct cells clusters and resemble regions of neuronal differentiation (regions 4 and 5 in Figure 2C) (Kantarci et al. 2015). The *frmpd4* expression domain co-localizes with *neurogenin1* (*neurog1*) at these stages, a key factor in otic neurogenesis (Supplementary Figure 5A) (Andermann et al. 2002). Knockdown of *neurog1* resulted in reduction of *frmpd4* expression in the otic vesicle and thereby indicate partial regulation of *frmpd4* by *neurog1* (Supplementary Figure 5B), while Morpholino knockdown of *frmpd4* did not result in prominent changes of *neurog1* or *isl1* expression patterns (Supplementary Figure 5C and 5D).

In summary, non-syndromic hearing loss and FRMPD4 expression patterns in hearing organs of vertebrates strongly implied a conserved molecular function during this process. To further investigate this, we conducted a study of hearing capacity in *frmpd4* loss of function models.

### FRMPD4 ortholog Ngh (CG42788) is required for proper hearing in *Drosophila melanogaster*

**Figure 3.**
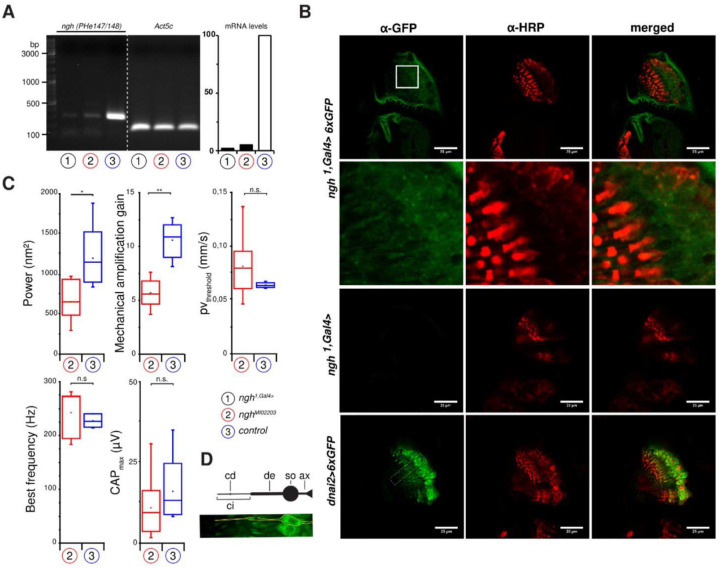
FRMPD4 function in hearing is evolutionary conserved in invertebrates. **(A)** RT-PCR analysis of *ngh* expression (*ngh*^*1,Gal4*^; marked in black) and ENU mutants (*ngh*^*MI02203*^; marked in red) in comparison to wild type controls (*w*^*1118*^; marked in blue). *Act5c* was used as reference. **(B)** Expression analysis of *ngh* utilizing a Promotor-trap generated via CRISPR (*ngh*^*1,Gal4*^) and hexameric cytoplasmic GFP (6xGFP) as reporter gene. Weak expression of *ngh* (*ngh*^*1,Gal4*>^6xGFP) could be detected in JONs (*dnai2>6xGFP* as positive control). Scale bars indicate 25μm. **(C)** Hearing was quantified by measuring the resonance frequency of the mechanical free fluctuations of the antennal sound receiver, the power of these free fluctuations, the nonlinear amplification gain provided by JON motility, and sound particle velocity required to evoke electrical JON compound action potentials (CAPs), and the maximum amplitude of these CAPs. Statistical significance was calculated with Mann-Whitney U tests. Asterisks indicate significant differences between genotypes (*n.s.=* not significant). **(D)** Schematic view of the morphology of mechanosensitive JO neurons: ax (axon), so (soma), de (dendrite), ci (cilium), cd (ciliary dilation).

In *Drosophila melanogaster*, the gene *CG42788* (Supplementary Figure 6), which we renamed to *nicht gut hörend (ngh)*, is the sole ortholog of vertebrate *frmpd4* (28% sequence identity and 44% sequence similarity). RNA levels in KO mutant flies revealed practically no residual expression of *ngh* (Figure 3A). The mutants were viable, and the gross morphology of their antennal ears appeared to be unaffected by the loss of *ngh*. Initial database screening of the *CG42788* (*ngh*) gene imply a rather broad expression pattern during developmental stages and in adult organs, e.g. adipose, reproductive and muscle system. Specific reporter gene analysis, using a hexameric cytoplasmic GFP and a *ngh*^*1,Gal4*>^ *promotor trap*, revealed a weak expression in Johnston’s organ neurons (JONs), the chordotonal mechanosensory neurons that mediate hearing in the fly (Albert and Gopfert 2015) (Figure 3B). To assess auditory function, we first measured the mechanical free fluctuations of the antennal sound receiver in the absence of acoustic stimuli. These free fluctuations are actuated by thermal bombardment and motile responses of JONs (Göpfert et al. 2005). Compared to the antennal receivers of controls, those of *ngh* KO flies fluctuated with a lower power (Figure 3C), signaling a reduction of JON motility. The fluctuations were tuned to the same resonant frequency in mutants and controls (242 and 232 Hz, respectively), and we next stimulated the flies with pure tones at that frequency, while varying the sound particle velocity (Göpfert et al. 2006). When we plotted the displacement of the antennal receiver against the particle velocity, we found that the compressive nonlinearity is more pronounced in controls than in *ngh* KO flies. This compressive nonlinearity reports mechanical amplification by JON motility, which nonlinearly boosts the mechanical sensitivity of the antennal receiver when sound is faint (Göpfert et al. 2006). Compared to controls, the gain of this mechanical amplification was reduced in *ngh* KO flies, signaling that loss of *ngh* impairs JON motility and active mechanical amplification in the ear. Along with these mechanical defects, slightly, though non-significantly larger sound particle velocities were required in controls to evoke compound action potentials of JONs in the mutants than in controls, and maximum CAP amplitudes were lightly reduced. Hence, hearing in *Drosophila* is modulated by *ngh*, the *Drosophila* ortholog of *frmpd4*.

### *frmpd4* KO in zebrafish causes inner ear and posterior lateral line defects

Expression of *frmpd4* in auditory organs and their precursor structures in zebrafish embryos imply a functional conservation or a comparable role to *FRMPD4* during hearing perception in humans. Sound perception in fish is different to hearing in higher vertebrates as anatomical adaptations to water habitats results in the detection of water vibrations and flow changes rather than sound waves (Nicolson 2005; Whitfield 2002). Specialized sensory cell clusters, e.g. neuromast cells of the lateral line organ, are thought to be orthologue structures to hair cells in the cochlea of higher vertebrates and function as mechanoreceptors of the auditory system (Atkinson et al. 2015; Dambly-Chaudière et al. 2003).

**Figure 4.**
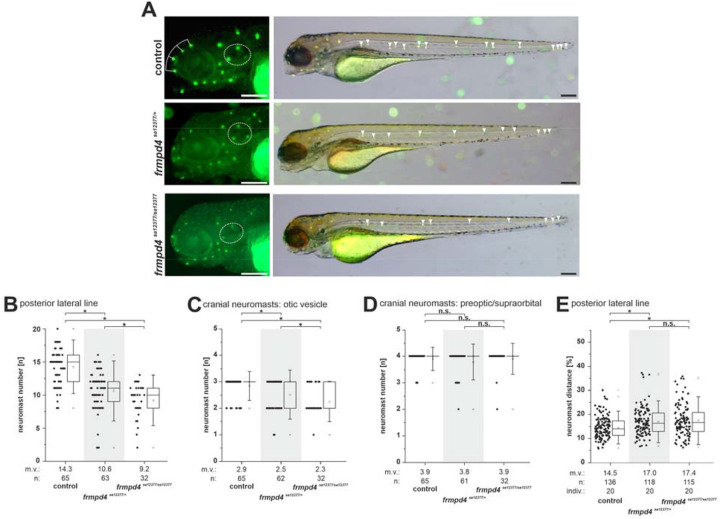
Loss of function of *frmpd4* in zebrafish results in reduced neuromast number and distance changes. **(A)** *frmpd4*^sa12377^ mutants display no general developmental defects, but show loss of DASPEI positive neuromast cells in the posterior otic vesicle (dashed white circle) and in the posterior lateral line (white arrowheads). Quantification of DASPEI positive neuromast cell number in the posterior lateral line **(B)** and the otic vesicle **(C)** showed significantly reduced amounts in heterozygous and even stronger reduction in homozygous *frmpd4* mutants. **(D)** Preoptic and supraorbital neuromast cells are not lost in the *frmpd4*^sa12377^ mutants (the corresponding cell cluster dorsal of the eye marked with white lines in A). **(E)** Measurements of relative distance between neuromasts in the posterior lateral line indicate increased relative distances between clusters in *frmpd4*^*sa12377*^ mutants. m.v.: mean value; n: data point amount; indiv.: number of investigated individuals; statistical significance was calculated by a two-tailed Mann-Whitney U test. An asterisk indicates significant changes between groups, while n.s. marks not significantly different groups. Scale bars in A indicate 100μm.

Investigations of *frmpd4*’s role during the development and function of hearing organs in zebrafish were performed by loss of function experiments (different zebrafish genetic tools are summarized in Supplementary Figure 7). We utilized splice site blocking Morpholinos (Supplementary Figure 8), transient F0 CRISPants (Supplementary Figure 9) and splice site deficient *frmpd4* ENU mutants (genomic feature: sa12377; Supplementary Figure 10) to determine the appearance of sensory neuromast clusters marked by the fluorescent vital dye DASPEI (2-(4-(dimethylamino)styryl)-N-ethylpyridinium iodide) (Harris et al. 2003). The *frmpd4*^sa12377^ variant leads to cDNA alterations, stabilized intron 10–11 and results in mis-splicing events (Supplementary Figure 7C), thereby disrupting normal Frmpd4 protein function. In general, reduction of *frmpd4* function resulted in reduction of detectable neuromast clusters in the PLL and in the otic vesicle (OV; Figure 4A). Along with the reduced amount of neuromast clusters in the posterior lateral line (Figure 4B) and in the otic vesicle (Figure 4C), the distance between the single clusters was increased (Figure 4E). Especially homozygous *frmpd4* mutants clearly showed these characteristics, with only an average of 6.64 clusters PLL/larvae (52.7% reduction to control) and an average of 2.00 clusters PLL/larvae (28.2% reduction to control) neuromast clusters in the PLL and the OV, respectively. The changes in the PLL resulted in a decrease of neuromast clusters and an increase of mean distance between the clusters of 2.94% average of relative distance between PLL clusters. Overall embryo and larval morphology was not influenced by *frmpd4* loss of function and neuromast clusters in regions of no *frmpd4* expression, like preoptic and supraorbital, did not show notable reduction in *frmpd4*^sa12377^ mutants (Figure 4D). In support of different genetic models, gain-of-function experiments of full-length FRMPD4 and of patient variants via RNA injection into zebrafish embryos resulted in changed neuromast numbers within the PLL, but did not significantly change other neuromast patterns like neuromast number in OV or neuromast distances (Supplementary Figure 10).

**Figure 5.**
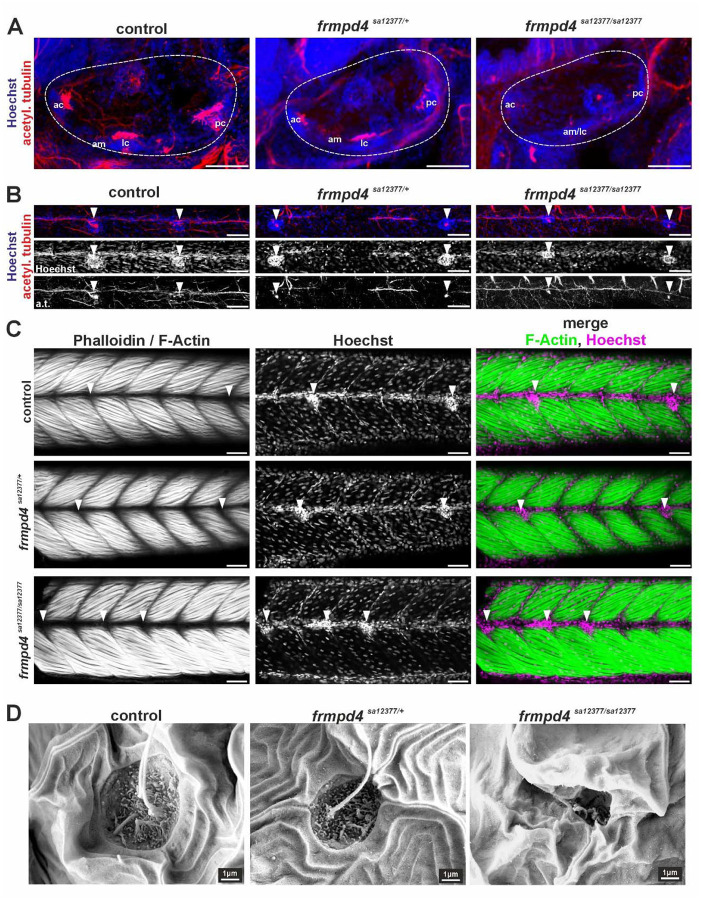
Loss of function of *frmpd4* in zebrafish results in axonal and structural malformations in the otic vesicle and the posterior lateral line. **(A)** Staining for acetylated tubulin in 4 dpf embryos indicated loss of neuronal cell and axonal projection in ventral sensory patches of the otic vesicle in homozygous *frmpd4*^*sa12377/sa12377*^ mutants. **(B)** Posterior lateral line neuromasts and axons (white arrowheads) are affected by *frmpd4* loss by depicting size reduction and morphological changes (analyzed embryos per genotype: 7 wild type controls; 6 heterozygous *frmpd4sa12377/+*; 5 homozygous *frmpd4sa12377/sa12377*). **(C)** Neuromast cell deposition in the PLL can be disrupted in homozygous *frmpd4*^*sa12377/sa12377*^ mutants, while adjacent somites show normal patterns (n=3 embryos per genotype; control and heterozygous *frmpd4*^*sa12377/+*^ mutants display indistinguishable patterns). **(D)** Scanning electron microscopy further showed disruption of cellular organization in PLL neuromasts and loss of kinocilia in in *frmpd4*^*sa12377/sa12377*^ mutants (n=3 per genotype). am: anterior macula, pm: posterior macula, ac: anterior crista, lc: lateral crista, pc: posterior crista. Scale bars in A to C indicate 50 μm.

To further investigate the neuronal and structural consequences of *frmpd4* loss on sensory patches in the OV and in the PLL, we performed immunofluorescence staining of acetylated tubulin in axons of 4 days post-fertilization (dpf) old fish larvae (Figure 5A and B; Supplementary Figure 8F and 8G). In the OV, this staining marks sensory patches corresponding to pseudostratified epithelium of the inner ear consisting of sensory hair cells and supporting cells, like the anterior and posterior macula, the anterior, posterior and lateral cristae (Haddon and Lewis 1996). Reduction of Frmpd4 function in heterozygous *frmpd4*^*sa12377/+*^ and most prominently in homozygous *frmpd4*^*sa12377/sa12377*^ mutants results in reduction of neuronal cell and axonal projection in these sensory patches, especially in the posterior cristae (Figure 5A). Neuromasts are deposited along the PLL and can be identified by their typical structure visible by nuclear staining. In accordance with the otic vesicle phenotype and the DASPEI staining (Figure 5B), PLL neuromasts are affected in *frmpd4*^*sa12377/sa12377*^ mutants. They depict reduced axonal outgrowth, reduction of cell nuclei in neuromasts and potentially a reduced lateral line nerve, as indicated by weaker acetylated tubulin signal (Figure 5B). To exclude structural changes caused by defects in surrounding somite muscles tissues, F-actin/Phalloidin staining was performed (Figure 5C). This experiment showed normal muscle development in controls and in *frmpd4*^*sa12377/sa12377*^ mutants, although localization of neuromasts is altered (indicated by white arrowheads). To investigate potential structural or anatomical changes in PLL neuromasts, we performed high magnification scanning electron microscopy (Figure 5D). While control or *frmpd4*^*sa12377/+*^ heterozygous individuals did not show severe morphological disruptions in PLL neuromasts, *frmpd4*^*sa12377/sa12377*^ individuals depict prominent disrupted structures, with reduced kinocilia and collapsed epidermal openings. Taken together, these results suggest a loss of hearing capacity in zebrafish after loss of Frmpd4 function due to either neurological or anatomical changes.

### Loss of Frmpd4 function in zebrafish results in reduced reactions to acoustic stimuli

**Figure 6.**
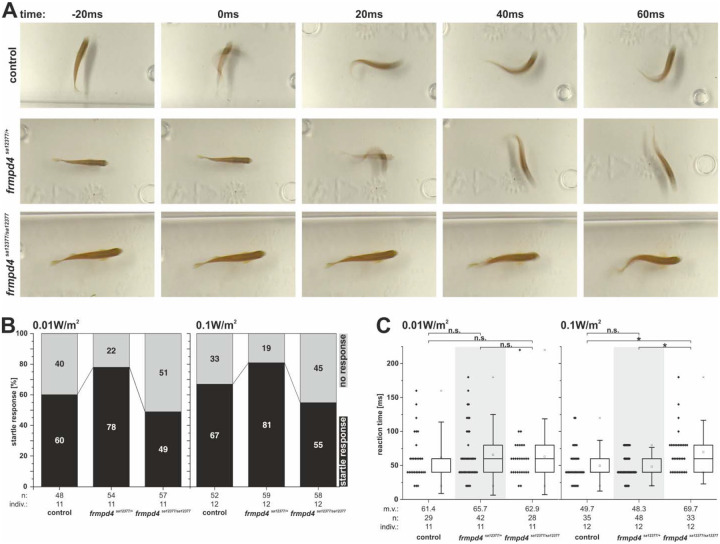
Loss of function of *frmpd4* in zebrafish results in reduced startle response behavior and slower reaction times. Startle response reaction to a given sound stimulus (sound levels in air low dB: 100 dB = 0.01 W/m^2^; high dB: >110 dB = 0.1 W/m^2^; 4400 Herz, 20 ms) is hampered in *frmpd4*^*sa12377/sa12377*^ mutants. **(A)** Single images from high-speed recordings of fishes reacting to a given sound stimulus (0ms). Quantification of startle responses **(B)** and reaction times **(C)** indicate reduced acoustic perception and slower response in *frmpd4sa12377/sa12377* mutants. m.v.: mean value; n: data point amount; indiv.: number of investigated individuals. Statistical significance as calculated by a two-tailed Mann-Whitney U test. Asterisks indicate significant changes between groups, while n.s. marks not significantly different groups. Whiskers indicate standard deviations (coefficient: 1.5).

Our zebrafish investigations indicated neuronal and anatomical alterations in zebrafish larvae and implied influence on sound perception and subsequently on behavior patterns. Normal zebrafish sound perception is fast and can result in an autonomous reflex called the “startle response”. This response represents an instinctive escape behavior to sudden, unexpected threats and stimuli (Colwill and Creton 2011; Zeddies and Fay 2005). We tested adult *frmpd4*^*sa12377*^ mutants in a hearing set up and quantified the appearance of startle responses and the response time after a given sound stimulus (Figure 6A; Supplementary Movie 1 and 2). WT and animals heterozygous for the *frmpd4*^*sa12377*^ allele reacted similarly to a given sound stimulus, while animals homozygous for the *frmpd4*^*sa12377*^ allele reacted at a much lower frequency (Figure 6B). Measurement of the reaction time further indicated that animals of the *frmpd4*^*sa12377/sa12377*^ group also reacted much slower to a given sound stimulus (Figure 6C).

### *Frmpd4*^−/−^ mice have reduced ABR amplitudes and high-frequency hearing loss

**Figure 7.**
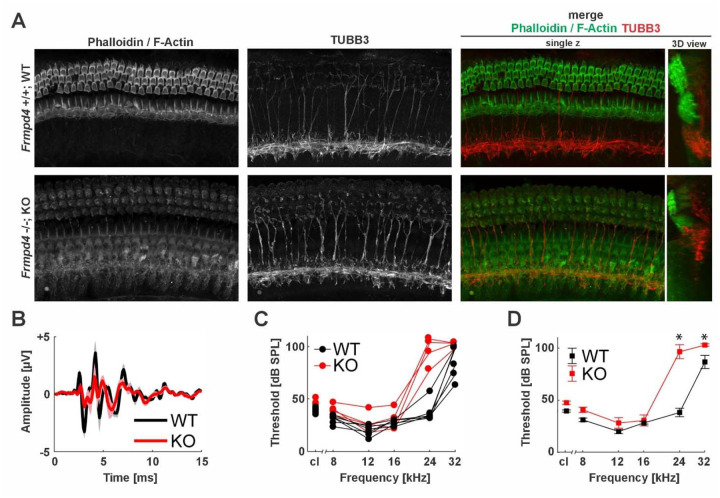
Knockout of *Frmpd4* displays morphological changes in the cochlea and elevates high-frequency ABR thresholds. **(A)** Confocal laser scanning images of mouse cochlear display altered epithelial structures in *Frmpd4* KO, visualized by top-down views and simultaneous Phalloidin (F-Actin) and TubB3 staining. **(B)** Grand average ABR waveforms in response to a click presented at 90 dB peSPL. Shaded regions indicate standard deviation of the mean. **(C)** Individual ABR thresholds in response to clicks (cl) and tones. **(D)** Mean ABR thresholds in response to clicks and tones. Error bars represent standard error of the mean. Asterisks indicate significant differences between genotypes.

Cochlear morphology was investigated in conditional KO mice, originally called *Preso1*^−/−^ in initial studies, hereafter referred to as *Frmpd4*^−/−^ mice, where they exhibited sustained, metabotropic glutamate receptor (mGluR) 5-dependent inflammatory pain linked to enhanced mGluR signaling (Hu et al. 2012). Although these mice were viable, fertile, and showed similar development and breeding behaviors as WT littermates, the cochlear epithelium of *Frmpd4*^−/−^ KO animals was morphologically altered in histological sections at level of the outer and inner hair cells visualized by F-Actin/Phalloidin localisation (Figure 7A). Simultaneously, unchanged TUBB3 expression was detectable in WT and KO animals and implies normal neuronal development in the organ of Corti. Subsequent functional measurements of hearing capacity imply that waveforms of *Frmpd4*^−/−^ are smaller with shifts in latency (Figure 7B). Individual (Figure 7C) and mean (Figure 7D) ABR thresholds with clicks and tones indicated high frequency hearing loss of *Frmpd4*^−/−^ mice, with significant differences seen at 24 (t(49)=11.6, p <0.0001) and 32 kHz (t(49)=3.2, p= 0.0134), but not other frequencies. In the mixed model, the interaction between genotype and frequency was significant (F(6,48)=17.2, p<0.0001), reflecting the observation that hearing loss in *Frmpd4*^−/−^ mice was limited to high frequencies.

## Discussion

The identification of genetic causes of non-syndromic hearing loss is important for accurate diagnosis, prognosis, and clinical management. We describe two families with maternally inherited *FRMPD4* missense variants with bilateral sensorineural non-syndromic hearing loss. Intellectual disability and other phenotypes such as epilepsy that have been attributed to *FRMPD4* variants were not observed in these individuals. Extensive annual or semi-annual medical follow-up of Family 1 for more than 20 years suggests that a developmental phenotype is unlikely. This work expands the phenotypic spectrum associated with disease-associated variants in *FRMPD4* and has implications for clinical management, molecular genetic diagnostic testing, genetic counseling, therapeutic decision-making, while also opening new avenues for studying mutational mechanisms and underlying pathophysiological processes.

To investigate molecular mechanisms linking *FRMPD4* to hearing loss, we analyzed its expression in auditory organs across several vertebrate species. Investigation of *Frmpd4* expression and protein localization in mouse and rat cochleae indicated general expression in neuronal tissues, with comparatively low expression levels in the cochlea. Further analysis of RNA sequencing data from the developing mouse cochlea showed evidence of increasing expression in the spiral ganglion neurons that is sustained into adulthood.

Subsequent protein localization further clarified specific FRMPD4 localization in organ of Corti and spiral ganglion during development (stage P1–P28), partly resembling the general spiral ganglion neuron marker TUBB3. Investigation of *frmpd4* expression in zebrafish embryos showed transcripts in early progenitor cells of corresponding hearing organs (OV and PLL). Although expression in zebrafish embryos is highly dynamic, sustained expression was identified in the OV in a pattern partly localized with *neurog1*, a marker for early neural differentiation (Korzh et al. 1998) and cranial sensory ganglia (Andermann et al. 2002; Yu et al. 2020).

The association between FRMPD4 and hearing appears to be evolutionarily conserved in *Drosophila*, where the FRMPD4 ortholog *ngh* is expressed in JONs, the auditory sensory cells of the fly. Loss of the *ngh* function impairs JON motility, resulting in reduced mechanical amplification during hearing, and decreased maximum amplitudes of sound-evoked CAPs of the JONs. The residual mechanical amplification and CAPs that persist in *ngh* KO flies imply that only a subset of the approximately 500 JONs in the *Drosophila* ear are functionally compromised in the KO flies, despite apparent *ngh* expression across all JONs. Follow-up studies will be required to clarify how *ngh* dosage contributes to JON function and to further assess conservation of auditory mechanisms across species.

Loss of function experiments in zebrafish, including splice site deficient ENU mutants, splice site blocking Morphants, and transient CRISPR/Cas9 genetic approaches, indicate a role for *frmpd4* in the development of auditory organs during embryonic and early larval development, as shown by reduced neuronal cell populations in both the OV and PLL. Additionally, morphological alterations in PLL neuromasts further support a structural role for Frmpd4 and suggest an influence on neuronal cell number. Migration of PLL primordium cells is not abolished following Frmpd4 reduction, as neuromasts are still detected along the entire body axis of affected embryos. Moreover, preliminary experiments indicate that expression of specific PLL markers *tfap2* and *cldnb* is preserved in *frmpd4* morphants (data not shown). Nevertheless, the reduced number of neuromast clusters, combined with fewer neuronal cells per neuromast is likely to impair function. Given that *frmpd4* expression was detected in the PLL primordium but not in neuromast cells, an early developmental effect of Frmpd4 on the PLL is plausible.

Consistent with these findings, hearing perception in *frmpd4* adult mutants is altered, as the startle response to sound stimulus is reduced and slower. This behavioral observation might be linked to additional *frmpd4* functions during neuronal development and cannot be separated from complex, higher brain function of adult individuals, but this phenotype mirrors a hearing-impaired situation similar to humans. Future studies will be required to determine whether these effects arise due to loss of PLL primordium cells, neuronal malformations, or result from morphological alterations of sensory organs. Recently, direct protein interaction between FRMPD4 and Whirlin-a (a protein associated with Usher syndrome type 2) was reported in the zebrafish retina, providing the first evidence for a potential postsynaptic protein complex involving FRMPD4 (Schellens et al. 2022). These findings hint to a neurological mode of Frmpd4 function mediated by interaction with Whirlin through C-terminal interaction via PDZ-binding motifs) and subsequent modulation of signaling via the GPSM2/LGN complex (Mauriac et al. 2017; Takayanagi et al. 2015; Yuzawa et al. 2011).

Remarkably, the mouse model presented here recapitulates the auditory phenotype observed in humans and argues against the presence of severe neurodevelopmental phenotypes previously associated with *FRMPD4*. Although a *Frmpd4*^*tm1dIcs*^ mouse line was previously characterized by the International Mouse Phenotyping Consortium (IMPC) and reported to have a non-significant ABR, acoustic startle, and pre-pulse inhibition results, these findings suggest that large-scale phenotyping pipelines focusing on young adult animals may lack sensitivity for detecting hearing impairment in older mice, particularly when higher frequencies are severely affected. Secondly, this mouse model was originally studied in the context of inflammatory pain without suspicion of an auditory phenotype (Hu et al. 2012), underscoring the importance of detailed and highly sensitive ABR testing. Behavioral audiogram thresholds may not be good predictors of ABR thresholds as the central auditory pathway can compensate for some peripheral dysfunction (e.g. central gain compensation).

The high-frequency hearing loss observed in *Frmpd4*^−/−^ mice is most similar to the phenotype observed in Family 1, where substantial residual hearing is present despite pan-frequency involvement. The comparatively more severe hearing loss in the proband of Family 2 remains unexplained, and we cannot exclude the contribution of genetic modifiers or additional undetected genetic variants contributing to these differences. Variable expressivity is common in hereditary hearing loss, including intrafamilial variability as observed in Family 1, where individual III:3 presented with moderate hearing loss and III:2 with mild hearing loss. Such variability may also have contributed to delayed recognition of hearing loss in the older sibling (III:2), who was diagnosed only retrospectively following newborn hearing screening and clinical diagnosis of his younger sibling.

**Figure 8.**
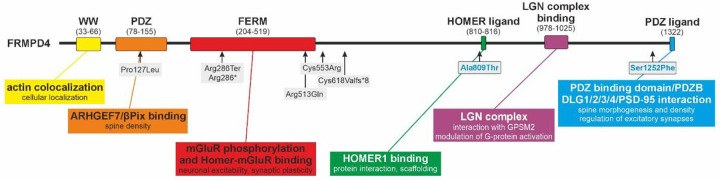
Overview of described FRMPD4 molecular functions linked to previously described variants and known protein domains. Newly reported genetic variants described in this study are located at the C-terminus and are highlighted in boxes with blue writing.

The patient variants described in this study are located at the C-terminal region of FRMPD4 and do not affect well-characterized functional domains at the N-terminus (see schematic overview in Figure 8). Protein structure prediction tools, such as by AlphaFold (Jumper et al. 2021; Varadi et al. 2022), perform poorly in these evolutionarily less conserved, intrinsically disordered regions and therefore do not provide reliable structural insight (UniProt: Q14CM0). Notably, the individuals reported here do not exhibit intellectual disability or epilepsy, in contrast to previously described pathogenic *FRMPD4* variants, including deletions or and truncating variants (listed in Supplementary Table 7). A previously reported patient with complete loss of the FRMPD4 N-terminus presented with X-linked intellectual disability and a dendritic spine density phenotype was functionally quantified (Piard et al. 2018), indicating a more severe neurological impact. This contrast suggests that the missense variants identified here do not result in complete loss of function, or that different FRMPD4 protein domains contribute to distinct phenotypes.

One hypothesis to be explored in future studies is that these newly associated *FRMPD4* missense variants with hearing loss interfere with neighboring protein domain interactions, leading to hearing loss without broader neurodevelopmental consequences. Candidate interactors include HOMER proteins, which share related functional features and exist in multiple transcript classes (Soloviev et al. 2000). *HOMER1* combines various molecular functions as it acts, on the one hand, as a scaffolding/multimodal adaptor protein (reviewed in (de Bartolomeis et al. 2022)). It further displays protein multimerization with CDC42 (Shiraishi-Yamaguchi et al. 2009), cytoskeletal organization during postsynaptic density to regulate homeostatic synaptic plasticity (Heavner et al. 2021). HOMER2 is associated with autosomal dominant non-syndromic hearing loss (DFNA68) and functional modeling in mice induces stereocilia abnormalities after over-expression and early-onset hearing loss after KO, respectively (Azaiez et al. 2015). We speculate that spatial-temporal interactions of FRMPD4 and HOMER proteins contribute as a key driver of diverse physiological outcomes. An alternative mechanism may involve FRMPD4 interaction with the LGN/GPSM2 adaptor protein complex via TPR-domain-mediated binding, as previously experimentally resolved for FRMPD1 (Takayanagi et al. 2015; Yuzawa et al. 2011). The LGN/GPSM2 complex is implicated in diverse cellular processes, including symmetric cell division, spindle orientation/localization, and neural stem cell division in the neuroepithelium (Bhonker et al. 2016; Konno et al. 2008; Mauriac et al. 2017). Disruptions of these pathways have been linked to hearing impairment through effects on actin-rich stereocilia elongation in auditory and vestibular hair cells, stereocilia row assembly, and regulation of actin dynamics in epithelial and neuronal tissues (Mauriac et al. 2017; Shi et al. 2022; Tadenev et al. 2019). Finally, the N-terminal PDZ domain of FRMPD4 has been shown to regulate dendritic spine morphology via interaction with PSD-95 (Lee et al. 2008) but has not yet been linked to hearing impairment. Nonetheless, PDZ domain-containing proteins are repeatedly implicated in auditory disorders, including Usher syndrome, where PDZ domain-containing scaffolding proteins such as WHRN (USH2D or DFNB31) orchestrate assembly of the USH2 complex (Stemerdink et al. 2022). Taken together, we speculate that the newly identified *FRMPD4* variants result in slight modulation of protein-protein interactions involving HOMER proteins, the LGN/GPSM2 complex, or PDZ-domain interactors, leading specifically to non-syndromic sensorineural hearing loss without broader neurodevelopmental manifestations.

## Conclusions

In summary, we identify *FRMPD4* as a novel X-linked gene for non-syndromic sensorineural hearing loss, expanding its previously described phenotypic spectrum beyond intellectual disability and epilepsy. Cross-species functional analyses in *Drosophila*, zebrafish, and mouse collectively establish an evolutionarily conserved role for FRMPD4 in auditory function, while the absence of neurodevelopmental features in affected individuals suggests that distinct protein domains and interaction networks underlie its diverse phenotypic manifestations. These findings have direct implications for molecular genetic diagnostics and variant interpretation in patients with hearing loss, and implicate *FRMPD4*-mediated interactions with HOMER proteins, the LGN/GPSM2 complex, and PDZ-domain partners as candidate mechanisms warranting investigation in future studies.

## Supplementary Material

Supplement 1

Supplement 2

1

## Figures and Tables

**Table 1.** *FRMPD4* variants identified in hearing impaired individuals.

F	ChrX Genomic location (g.)	*FRMPD4* c. position	FRMPD4 p. position	Zyg	AF gnomAD (v.4.1.0)	MAF gnomAD (v4.1.0)	MAF Pop gnomAD (v4.1.0)	TOPMed (v8)	All of Us	SIFT	PP-2	FATHMM	MT	REVEL	ClinPred	CADD
1	g.12718581C>T	c.3755C>T	p.(Ser1252Phe)	Hemi	NP	NP	NP	NP	NP	D	PrD	NS	D	U	D	23.90
2	g.12716884G>A	c.2425G>A	p.(Ala809Thr)	Hemi	1.7e-6*	2.2e-6*	European (non-Finnish)	NP	3.0e-6*	D	PrD	NS	D	U	D	25.00

All variants are annotated according to NC_000023.11 (genomic, GRCh38) and NM_001368397.1 (coding DNA). Residue position is according to NP_001355326.1. Abbreviations: AF, allele frequency; B, benign; chr, chromosome; D, Deleterious; F, family; MAF, maximum allele frequency; MT, MutationTaster; NP, not present; NS, not scored; Pop, population; PP2, PolyPhen-2; PrD, probably damaging; T, tolerated; U, uncertain; Zyg, zygosity.

* Identified in females

## Data Availability

In addition to the supplementary files, essential raw data files linked to this paper have been deposited at the Zenodo digital repository and are freely available for download here: **10.5281/zenodo.18507823** The repository data includes quantification tables for RT-qPCR experiments, quantification tables for zebrafish DASPEI experiments, mouse and *Drosophila* hearing measurements, additional images not used for figures and overview figures, specification of zebrafish hearing set-up and high-speed movies used for startle response quantification. Additional data supporting this study’s findings are available from the corresponding author upon reasonable request.

## List of abbreviations

ABR: auditory brainstem response
ax: axon
cd: ciliary dilation
ci: cilium
cl: clicks
DASPEI: 2-(4-(dimethylamino)styryl)-N-ethylpyridinium iodide
de: dendrite
dpf: days post-fertilization
FRMPD4: FERM and PDZ Domain Containing 4
gDNA: genomic DNA
HL: hearing level
hpf: hours post-fertilization
IMPC: International Mouse Phenotyping Consortium
indiv: number of investigated individuals
JO: Johnston’s organ
JONs: Johnston’s organ neurons
KO: knockout
m.v.: mean value
mGluR: metabotropic glutamate receptor
n: data point amount
ngh: nicht gut hörend
n.s.: not significant
OV: otic vesicle
PLL: posterior lateral line
PTA: pure-tone average
so: soma
WT: wild type
