# Supplementary material for: FRMPD4, a causal gene for intellectual disability and epilepsy, is associated with X-linked non-syndromic hearing loss
Source: medRxiv. 2026 Mar 30:2026.03.27.26349271. Preprint. [Version 1] doi: 10.64898/2026.03.27.26349271 (PMC13060437; doi:10.64898/2026.03.27.26349271)
Supplement: 1 [file NIHPP2026.03.27.26349271V1-supplement-1.pdf]

medRxiv preprint doi: https://doi.org/10.64898/2026.03.27.26349271; this version posted March 30, 2026. The copyright holder for this preprint (which was not certified by peer review) is the author/funder, who has granted medRxiv a license to display the preprint in perpetuity.
It is made available under a CC-BY 4.0 International license .

Supplemental Materials and Methods

Clinical evaluation and human genetics methods

Clinical assessment

Exome data filtering, analysis, and variant prioritization

***Drosophila* methods**

Animal models

RT-PCR

Immunofluorescence

Electrophysiological and mechanical recordings of the *Drosophila* ear

Zebrafish methods

Zebrafish whole mount *in-situ* hybridization

Zebrafish injections, Morpholino knockdown and CRISPR/Cas9 gene editing

Zebrafish genotyping

Scanning electron microscopy

DASPEI staining and neuromast quantification in zebrafish larvae

Immunofluorescence in zebrafish experiments

Confocal microscopy and imaging processing

Zebrafish startle response test

In vivo RNA overexpression

Mouse methods

RT-qPCR analysis of *Frmpd4* expression in mouse tissues

In silico analysis of *Frmpd4* expression in the cochlea and spiral ganglion neurons (SGNs) in the WT mouse

Immunofluorescence in mouse experiments

Auditory brainstem response (ABR) testing in mice

ABR regression analysis

medRxiv preprint doi: https://doi.org/10.64898/2026.03.27.26349271; this version posted March 30, 2026. The copyright holder for this preprint (which was not certified by peer review) is the author/funder, who has granted medRxiv a license to display the preprint in perpetuity.
It is made available under a CC-BY 4.0 International license .

Supplementary Tables

Supplementary Table 1. Primers used in the study

Supplementary Table 2. Exome filtering details for Family 1

Supplementary Table 3. Heterozygous variants identified in the affected individuals in Family 1 that were inherited from a healthy parent

Supplementary Table 4. Variants in known hearing loss genes and candidate genes identified in affected individuals through exome sequencing

Supplementary Table 5. Exome filtering details for Family 2, individual IV:1

Supplementary Table 6. Homozygous variants identified in individual IV:1 from Family 2

Supplementary Table 7. *FRMPD4* variants with clinical summary

Supplementary Figures

Supplementary Figure 1. Schematic overview of *FRMPD4* protein domain structure and disiease associated genetic variants reported to date

Supplementary Figure 2. Evolutionary conservation of Frmpd4 orthologues

Supplementary Figure 3. Summary of FRMPD4 domains and domain conservation in orthologues

Supplementary Figure 4. Additional mouse expression data

Supplementary Figure 5. *frmpd4* expression partly localizes with *neurog1* in the otic vesicle and is regulated by *neurog1*

Supplementary Figure 6. Overview of the *Drosophila* CG42788-RB gene locus and sound perception quantification

Supplementary Figure 7. Summary of Frmpd4 domains in zebrafish

Supplementary Figure 8. Loss of function of *frmpd4* in zebrafish results in neuromast alterations in the otic vesicle and in the posterior lateral line

Supplementary Figure 9. *frmpd4* CRISPant in zebrafish show only mild neuromast alterations in the otic vesicle and in the posterior lateral line.

Supplementary Figure 10. *FRMPD4* variant expression in zebrafish via RNA injection.

Supplementary Movies

Supplementary Movie 1: Examples of a startle response reaction to given sound stimulus, one individual per *frmpd4*^{sa12377} genotype, low dB sound stimulus (Frmpd4 lowbD.mp4)

medRxiv preprint doi: https://doi.org/10.64898/2026.03.27.26349271; this version posted March 30, 2026. The copyright holder for this preprint
(which was not certified by peer review) is the author/funder, who has granted medRxiv a license to display the preprint in perpetuity.
It is made available under a CC-BY 4.0 International license .

Supplementary Movie 2: Examples of a startle response reaction to given sound stimulus, one individual per *frmpd4*^{sa12377} genotype, high dB sound stimulus (Frmpd4 highbD.mp4)

medRxiv preprint doi: https://doi.org/10.64898/2026.03.27.26349271; this version posted March 30, 2026. The copyright holder for this preprint (which was not certified by peer review) is the author/funder, who has granted medRxiv a license to display the preprint in perpetuity.
It is made available under a CC-BY 4.0 International license .

Supplemental Materials and Methods

Clinical evaluation and human genetics methods

Clinical assessment

Diagnosis of sensorineural hearing loss was achieved according to current clinical standards and applied age-appropriate methods to determine hearing thresholds at routinely measured frequencies. Hearing loss threshold descriptions followed GENDEAF standards (Mazzoli et al. 2003). Routine pure-tone audiometry was performed on individuals III:2 and III:3 in Family 1 and III:2, and IV:1 of Family 2 according to current standards and measured hearing thresholds at 0.25, 0.5, 1, 2, 4, and 8 kHz. Air- and bone-conduction thresholds were measured and severity of hearing loss was determined by averaging pure-tone thresholds over 0.5, 1, 2 and 4 kHz (pure-tone average, $PTA_{0.5\text{-}4K}$). Otoacoustic emissions neonatal hearing screening and auditory brainstem response (ABR) testing was performed in individual III:3 of Family 1. Transient evoked otoacoustic emissions and distortion products were further tested in III:2, III:3 (Family 1) and IV:1 (Family 2). Auditory steady-state response was performed in IV:1 (Family 2). Tympanometry was performed in the affected individuals in Family 2. The severity of hearing loss in the better ear was defined as mild for thresholds averaging 20-40 dB hearing level, moderate for 41-70 dB hearing level, severe for 71-95 dB hearing level, and profound in excess of 95 dB hearing level. Progressive hearing loss was defined as a deterioration of >15 dB hearing level in the average over the frequencies of 0.5, 1, and 2 kHz within a 10-year period.

Exome data filtering, analysis, and variant prioritization

Exome data were filtered through pedigree-based strategies to evaluate autosomal recessive, autosomal dominant, and X-linked modes of inheritance. The bioinformatics filtering strategy focused on exonic and donor/acceptor splicing variants as previously described (Vona et al. 2021). Alternative alleles present at >20% and a minor allele frequency <0.01 were assessed using gnomAD v4.1.0 (Chen et al. 2024), TopMed freeze.8 (Taliun et al. 2021), and data from the All of Us research program (The All of Us Research Program Genomics Investigators et al. 2024). MAF thresholds were later adjusted according to ACMG/AMP guidelines for hearing loss (Oza et al. 2018) and an in-house epilepsy cohort (n=511) with a threshold of ≤1%. Artifact-prone gene families (*HLA*s, *MAGE*s, *MUC*s, *NBPF*s, *OR*s, *PRAME*s) were excluded. Variant types that were analyzed included SNVs, indels, and splicing variants with potential structural or cryptic splice site effects. Variants in 5' and 3' UTRs, deep intronic, and intergenic regions were excluded. Allele read frequencies between 25-75% were classified as heterozygous; those >75% as homozygous. Clinical databases ClinVar (Landrum et al. 2025) and the Deafness Variation Database (DVD) (Azaiez et al. 2018) were referenced to assess already reported interpretations.

medRxiv preprint doi: https://doi.org/10.64898/2026.03.27.26349271; this version posted March 30, 2026. The copyright holder for this preprint (which was not certified by peer review) is the author/funder, who has granted medRxiv a license to display the preprint in perpetuity.
It is made available under a CC-BY 4.0 International license .

Pathogenicity of variants was assessed using multiple *in silico* tools: SIFT (Schwarz et al. 2014), PolyPhen-2 (Adzhubei et al. 2010), FATHMM (Shihab et al. 2013), MutationTaster (Steinhaus et al. 2021), REVEL (Ioannidis et al. 2016), ClinPred (Alirezaie et al. 2018), and CADD (Schubach et al. 2024). Computational assessment of splicing effects used SpliceSiteFinder-like, MaxEntScan, NNSplice, and GeneSplicer embedded in Alamut Visual Plus v1.12 (Sophia Genetics, Bidart, France) as well as SpliceAI Visual and AbSplice embedded in SpliceAI Visual (De Sainte Agathe et al. 2023). Residual Variation Intolerance Score (RVIS) was used to assess gene intolerance to functional variants (Petrovski et al. 2013).

Drosophila methods

Animal models

Fly (*Drosophila melanogaster*) stocks used in this study and their respective origin were *ngh*^{MI02203} (BDSC 60756) mutants and *w*^{1118} (BDSC 3605) controls. For expression analysis, the Gal4/UAS-system (Brand and Perrimon 1993) was utilized, with *dnai2-Gal4* (Karak et al. 2015) and hexameric GFP (BDSC 52261).

Targeted deletion and Gal4 knock-in at the *CG42788* (*ngh*) locus were achieved using CRISPR/Cas9-mediated genome editing. Guide RNAs were designed with the Target Finder tool (https://flycrispr.org/) and cloned into the vectors pBFv-U6.2 (Addgene #138400) and pBFv-U6.2B (Addgene #138401). Both gRNA cassettes were subsequently combined into a single construct via *NotI* and *XhoI* restriction sites. For the donor construct, a 1032 bp fragment upstream of the *ngh* transcriptional start site was cloned into the phD-DsRed vector (Addgene #51434), followed by insertion of the *Gal4* coding sequence derived from the pt-Gal4 plasmid (Sharma et al. 2002). Downstream of the 3×P3-DsRed selection marker, an 843 bp 3′ homology arm corresponding to the *ngh* locus was inserted, yielding the final plasmid phD-DsRed-*ngh*^{upstream}-Gal4-3×P3>DsRed- *ngh*^{Downstream}. The donor and gRNA plasmids were co-injected into embryos of *vas-Cas9*(II) flies (BDSC #56552). Correct deletion and targeted integration of *Gal4* at the *ngh* transcription start site were verified by DsRed reporter fluorescence in the eyes and confirmed by sequencing of the edited genomic locus.

Flies were kept at 25°C, 60% humidity on standard cornmeal-yeast medium in a 12-h/12-h light/dark cycle. Experiments were performed in accordance with German Federal regulations (license Gen.Az 501.40611/0166/501).

RT-PCR

medRxiv preprint doi: https://doi.org/10.64898/2026.03.27.26349271; this version posted March 30, 2026. The copyright holder for this preprint (which was not certified by peer review) is the author/funder, who has granted medRxiv a license to display the preprint in perpetuity.
It is made available under a CC-BY 4.0 International license .

Three independent cohorts, each consisting of 20 fly heads per genotype, were snap-frozen in liquid nitrogen for RNA extraction. Total RNA was isolated using the ZR Tissue & Insect RNA MicroPrep Kit (Zymo Research Europe GmbH, Freiburg, Germany; #R2030). 1 µg of RNA per sample was reverse-transcribed into cDNA using the QuantiTect Reverse Transcription Kit (Qiagen, Valencia, CA, USA; Cat. No. 205311). For amplification, 10 ng of cDNA was used per reaction (technical triplicates) with the Phire Tissue Direct PCR Master Mix (Thermo Fisher Scientific; F170S) and *ngh*-specific primers, using *Act5C* as the reference gene. Cycling conditions were: 95°C for 5 min, followed by 40 cycles of 95°C for 15 s, 54°C for 30 s, and 72°C for 30 s, with a final extension at 72°C for 10 min. PCR products were separated by agarose gel electrophoresis, stained with ROTI®GelStain (Carl Roth), and visualized using an iBright gel documentation system (Thermo Fisher Scientific). Band intensities were quantified using the iBright analysis software.

Immunoflourescence

GFP expression was visualized in fixed adult Johnston's organ (JO) tissue sections. Staining was performed as described (Hehlert et al. 2025). In brief, fly 2nd antennal segment sections were prepared from five-day adult fly heads. Heads of adult flies, five days post-eclosure, were isolated and fixed in 4% PFA (Merck) for 1 h at room temperature. After a brief wash with 1x PBS (Merck, P4417) heads were embedded in albumiSn gelatine and fixed with 6% PFA in 0.3% PBS-T (Triton X100, Sigma-Aldrich), pH 7.4, at 4°C overnight. The embedded samples were then fixed in fresh 100% methanol (Merck) for 10 min at 4°C and quickly transferred to 1x PBS for re-hydration. 40 µm tissue sections were generated with a microtome, and tissue slices were stored in 1x PBS-T. Blocking was done in blocking buffer (1x PBS-T, 5% normal goat serum, 2% BSA) for 1h at room temperature. Tissues were then incubated with FluoTag®-X4 Atto 488nm anti-GFP (1:1000, NanoTag Biotechnologies, N0304) and Cy3-conjugated goat anti-HRP (1:300, Jackson ImmunoResearch, 123-165-021) in blocking buffer for 2 h at room temperature. Afterwards samples were washed three times in 1x PBS-T (10min, room temperature). Tissue samples were then mounted on microscope slides in DABCO (Carl Roth, 0718.1). Stainings were analyzed with a Leica SP8 microscope (at 20°C) in 8-bit mode using a C-Apochromat 63x/1.40 W Korr FCS M27 objective and the Leica X software. For fluorescence detection, the following settings were used: Atto488 / Alexa-488 (Ex: 488nm; Em: 490–540 nm); Cy3 (Ex: 561nm; Em: 566–600 nm). Images were subsequently processed in Image J v1.49m (NIH) and arranged in Adobe Illustrator CC.

Electrophysiological and mechanical recordings of the *Drosophila* ear

Recordings of antennal mechanics and Johnston's organ neurons (JON) compound action potentials (CAPs) were performed as described (Senthilan et al. 2012). Single flies were completely immobilized ventral side down in paraffin:beeswax (50:50), leaving only the

medRxiv preprint doi: https://doi.org/10.64898/2026.03.27.26349271; this version posted March 30, 2026. The copyright holder for this preprint (which was not certified by peer review) is the author/funder, who has granted medRxiv a license to display the preprint in perpetuity.
It is made available under a CC-BY 4.0 International license .

recorded antenna and arista unobstructed. Antennal vibrations were measured at the arista tip using a laser Doppler vibrometer (PSV-400, Polytec, Waldbronn). Acoustic pure tone stimuli were broadcast with a loudspeaker (Visaton W130S) positioned 10 cm behind the animal. Sound particle velocity at the position of the fly was monitored with a pressure-gradient microphone (Emkay NR3158; Knowles Electronics).

Microphone, laser, and electrode signals were simultaneously digitized at 8.2 kHz. Recordings were divided into 1 s segments (rectangular window function) and subjected to discrete Fast Fourier transformation (FFT). 40-70 segments were averaged for determining power spectra of the mechanical fluctuations of the antennal sound receiver in the absence of acoustic stimulation, and ten segments were averaged to assess pure tone-induced responses of the antenna and the JONs.

Power spectra of mechanical free fluctuations were fitted with a Harmonic oscillator function to determine the resonance frequency (best frequency) of the antennal sound receiver (Göpfert et al. 2005). The respective fluctuation power was assessed by integrating the power spectrum for frequencies between 150 and 1,500 Hz. Mechanical sensitivity of the sound receiver was determined from its displacement response to pure tone stimulation at its resonance frequency, by dividing its displacement by the corresponding sound particle velocity. The mechanical amplification gain provided by JON motility was quantified by dividing the mean mechanical sensitivity of the receiver in the low-intensity linear regime by that in the high-intensity linear regime.

During tonal stimulation, Fourier amplitudes of the microphone and laser signals were extracted at the stimulus frequency, whereas CAP amplitudes were quantified at twice that frequency to account for the characteristic frequency doubling of the JO response. Particle velocity (pv) thresholds of the CAPS were determined as the minimum particle velocity required to elicit ≥10% of the normalized CAP response amplitude (Senthilan et al. 2012).

Zebrafish methods

Zebrafish whole-mount *in situ* hybridization

RNA *in situ* hybridization was performed according to standard protocols (Thisse and Thisse 2008). RNA probes were synthesized from cloned partial mRNA sequences (newly cloned *frmpd4* probe included exon 8 to 12 to target FERM domain sequences; *neurog1* (Blader et al. 1997) and *isl1a* (Appel et al. 1995) probes were previously published) of target genes using the DIG or FLU RNA Labeling Kit (Roche). Sense probes were synthesized as a negative control for each anti-sense probe and used under the same reaction conditions. Specific staining was additionally compared to expression patterns deposited in zfin.org. For signal improvement, embryos were incubated after staining in 100% methanol over night at -

medRxiv preprint doi: https://doi.org/10.64898/2026.03.27.26349271; this version posted March 30, 2026. The copyright holder for this preprint (which was not certified by peer review) is the author/funder, who has granted medRxiv a license to display the preprint in perpetuity.
It is made available under a CC-BY 4.0 International license .

20°C prior to rehydration in PBST, mounting and imaging. Embryos were glycerol mounted, viewed either with a Leica S8 APO Stereomicroscope and were photographed using a Leica MC170HD digital camera or a Zeiss Imager A1 and were photographed using a Zeiss MRc5 digital camera. Digital pictures were acquired via the Leica LAS or the Zeiss Axiovision software and arranged using Corel Draw X6 graphics suite.

Zebrafish injections, Morpholino knockdown and CRISPR/Cas9 gene editing

One-cell to maximum four-cell stage zebrafish embryos were injected with solutions comprising of an active reagent (e.g. RNA, DNA or Morpholino), Phenol red (pH 7.0; 0.05% final concentration; for visualization of injection solution) and Fluorescein-Isothiocyanate-Dextran (FITC; Sigma-Aldrich; 1 mg/µl; for visualization of successful and uniform injection in 24 hpf embryos). Positively injected embryos were identified 24 hpf by transient green FITC fluorescence and were further analyzed. In general, injection volumes are adjusted to 1/10 volume of the first cell and were controlled by measurements of droplet size in mineral oil on a micrometer scale slide followed by volume calculation (V = 1/6πd3; mean droplet volume: ~8 nl).

Antisense Morpholino oligonucleotides were synthesized by GeneTools (Philomath, OR, USA). The *frmpd4* splice site Morpholino 5´-TACACCTGTGTGCCACAAAGAGACA-3´ targeted the exon-intron boundary of zebrafish *frmpd4* intron 14-15 and exon 15 (ENSDARE00000872050). The Morpholino is stabilizing an unspliced product with an addition of 330 bp, which is leading to a premature stop codon. The neurog1 Morpholino 5´-ACGATCTCCATTGTTGATAACCTGG-3´ targeted the start codon of *neurog1* and has been previously published (Andermann et al. 2002). A standard control oligo Morpholino 5´-CCTCTTACCTCAGTTACAATTTATA-3´ was injected in equal amounts as a negative control to exclude unspecific effects. Unless otherwise noted, a 0.25 mM Morpholino solution was injected into each embryo.

Injection of two sgRNAs targeting two positions in the zebrafish *frmpd4* gene locus (sgRNA r2 and sgRNA f3; final RNA concentration each 25ng/µl), along with nCas9n RNA (final RNA concentration 150ng/µl) (Jao et al. 2013) results in transient F_0 CRISPants. Application of this method results in mosaic embryos possessing deletion of a 199 bp gDNA fragment, flanking the conserved Serine position associated with hearing loss in Family 1. Functionality of used sgRNAs and Cas9 RNA was assessed by sequencing gDNA of eight injected embryos 4-5 dpf for locus specific alterations. Primer sequences are shown in Supplementary Table 1.

Zebrafish genotyping

Extraction of genomic DNA from fin clips or from whole 4-5 dpf embryos was performed by Proteinase K digestion as previously described (Westerfield 2000). 2 µl of eluted genomic

medRxiv preprint doi: https://doi.org/10.64898/2026.03.27.26349271; this version posted March 30, 2026. The copyright holder for this preprint (which was not certified by peer review) is the author/funder, who has granted medRxiv a license to display the preprint in perpetuity.
It is made available under a CC-BY 4.0 International license .

DNA (approx. 50 ng/µl) was used for PCR amplification, with subsequent clean-up and Sanger sequencing. Sequences of primers used in this study are given in Supplementary Table 1. Sequencing results were analyzed with "ApE" (http://biologylabs.utah.edu/jorgensen/wayned/ape/) and CodonCode Aligner (CodonCode Corporation, Centerville, MA, USA) software packages.

Scanning electron microscopy

Zebrafish larvae at 5 dpf were fixed overnight at 4°C with 6.5% glutaraldehyde solution in Sörens buffer. After fixation, embryos were washed 3x in PBS and dehydrated by an uprising dilution series into acetone. Subsequently, embryos underwent critical point drying and were gold/palladium coated. Images were taken with a JEOL JSM-7500F (Akishima, Tokyo, Japan), using either LEI or SEI detectors at 10,000x magnification.

DASPEI staining and neuromast quantification in zebrafish larvae

For hair cells and neuromast visualization in living larvae, the fluorescent dye 2-[4-(dimethylamino)styryl]-N-ethylpyridinium iodide (DASPEI; Life Technolgies, Carlsbad, CA, USA) was used. Larvae at the desired stage (age before 120 hpf) were incubated for 15 minutes in a 0.13 mM DASPEI solution in 30% Danieau's medium. The larvae were subsequently washed three times for one minute in 30% Danieau's. Finally, a Tricain incubation was performed prior to imaging. The fish were mounted into a 2.5% methylcellulose/30% Danieau's medium solution and laterally imaged under a fluorescence stereomicroscope using light-field, gfp and rfp filters. Larvae of *frmpd4*^{sa12377} strain crossings were of unknown genotype during imaging and were subsequently fixed for postmortem genotype determination.

For the quantification of the number and distance between the neuromasts of the posterior lateral line (PLL, Clusters P1-P9) in images of DASPEI stained zebrafish Fiji software was used. Distances between clusters were measured in pixels. The resulting values of the pixel distances between the neuromasts were scaled to the overall length between P1 and P9 using Microsoft excel and thus representing the distances as percent values. The calculated relative distances were statistically analyzed using OriginPro 2021 and illustrated via boxplot diagrams including data points. For standard deviation, the coefficient value was set to 1.5 and is indicated by the whiskers within the blot. Further values depicted in the box blots are the median (parallel line), the mean value (small box), and the upper and lower quartile (large box). For statistical analysis, the obtained data values were first tested for normal distribution, and the significance was determined using the Mann-Whitney-U test. The significance values were given as U values with a significance level set to be smaller than 5 % ($U < 0.05$). Values with a statistical significance of $U \leq 0.05$ were marked with *, while a significance level of $U \leq 0.0001$ is marked as ** and *** for $U \leq 0.00001$.

medRxiv preprint doi: https://doi.org/10.64898/2026.03.27.26349271; this version posted March 30, 2026. The copyright holder for this preprint (which was not certified by peer review) is the author/funder, who has granted medRxiv a license to display the preprint in perpetuity.
It is made available under a CC-BY 4.0 International license .

For quantification of neuromast numbers in the laterally visible otic vesicle (OV) of the same embryos, DASPEI stained neuromast cells within the organ were manually counted subsequently. Quantification of preoptic/supraorbital neuromast clusters (ventral to the eye; indicated in control embryo Figure 3A) was performed as a control for normal cranial neuromast development. Statistical analyses were performed like PLL measurements using the Mann-Whitney-U test.

Immunofluorescence in zebrafish experiments

Immunofluorescence staining of axons and kinocilia in hair cells was performed according to previously established protocols (Raible and Kruse 2000; Tanimoto et al. 2011) using an acetylated Tubulin antibody (primary monoclonal mouse antibody IgG2b 6-11B-1; sc-23950, Santa Cruz Biotechnology, Antibody Registry nr. AB_628409; 1:1000 diluted in 1% sheep serum/PBST) and anti-mouse IgG Alexa 594 secondary antibody (A-1062; Thermo Fisher Scientific, Waltham, MA, USA; Antibody Registry nr. AB_2534109; 1:5000 diluted in PBST) or anti-mouse IgG Alexa Fluor 488 (A-11001; Thermo Fisher Scientific, Waltham, MA, USA; Antibody Registry nr. AB_2534069, 1:5000 diluted in PBST). For counterstaining of nuclei Hoechst 33342 (dilution 1:10000; Invitrogen/Thermo Fisher Scientific, Waltham, MA, USA) was added to the to the secondary antibody solution. F-Actin was visualized with Phalloidin-488 (1:20 dilution in PBST; Acti-stain 488, Biozol, Eching, Germany). Images were taken with a Nikon confocal laser scanning microscope A1+ and analyzed with Fiji/ImageJ software (https://fiji.sc/).

Confocal microscopy and imaging processing

High-resolution immunofluorescence images were taken with a Nikon confocal laser-scanning microscope A1+ setup with a D-eclipse C1si detector and corresponding SP fluorescence filters (Nikon) used for 405nm, 488nm, 561nm excitation wavelength lasers (Nikon Europe B.V.; Amstelveen, The Netherlands). For visualization of cranial or trunk region of zebrafish embryos, head and trunk were manually separated and individually mounted for confocal imaging. Standard confocal imaging utilized a Plan Apo VC 20x DIC N2 objective and recordings at 2048x2048 x/y-pixel size. Cranial region images included up to 55 z-stack images (overall imaging depth ~110µm), while trunk region images included up to 30 z-stack images (overall imaging depth ~60µm). Voxel size was set to 0.16x0.16x2.5µm up to 0.16x0.16x5µm. Acquired.nd2 files from NIS-Elements software (Nikon) were further processed with imageJ/Fiji. For visualization of neuromast centers in the otic vesicle or in the lateral line maximum intensity projections of ROIs were calculated. “Despeckle” routine was used to reduce image noise of confocal stack images. Color overlay images were processed for optimal orientation, brightness, contrast and were colored-coded by different LUTs to get best visualization.

medRxiv preprint doi: https://doi.org/10.64898/2026.03.27.26349271; this version posted March 30, 2026. The copyright holder for this preprint (which was not certified by peer review) is the author/funder, who has granted medRxiv a license to display the preprint in perpetuity.
It is made available under a CC-BY 4.0 International license .

Zebrafish startle response test

Startle response tests have been conducted as previously reported (Bhandiwad et al. 2013; Wang et al. 2015). In short, single adult zebrafish (aged between 5 and 7 months, mixed sex ratios) were investigated in an experimental set up and response to a given sound stimulus (frequency: 4.4 kHz; duration: 20ms; sound-intensity levels in air: 0.01W/m^2 (100dB) to 0.1W/m^2 (>110dB): 4 to 6 sound pulses in a 2 minute time frame) was digitally recorded after habituation with a high speed camera at 50 frames/second. Release of a startle response within a timeframe of 200 ms (10 frames) after the sound stimulus and reaction time was quantified (Software: VLC media player, Adobe Premier Elements 14 and ImageJ/Fiji). Reaction time was quantified by measuring frames between sound stimulus detection by the sound meter and animal reaction. Two independent experiments were conducted and overall, 12 fish per genetic group (wild type (WT); *frmpd4*$^{sa12377/+}$; *frmpd4*$^{sa12377/sa12737}$) were investigated.

In vivo RNA overexpression

RNA overexpression in zebrafish embryos was performed by microinjections of human *FRMPD4* capped mRNA (final concentration 25 ng/µl in injection solution) into one-cell stage zebrafish embryos. Injection controls were performed, by substituting mRNA with water. To produce WT and patient-specific *FRMPD4* capped mRNA, full-length *FRMPD4* CDS (Dharmacon Clone-ID 8322752; Accession nr.: BC113700; Horizon Discovery) was subcloned into pCS2p+ (pCS2P+ was a gift from Marc Kirschner; Addgene plasmid #17095; http://n2t.net/addgene:17095; RRID:Addgene 17095). Patient-specific variants were introduced via site-directed mutagenesis (NEB Q5® Site-Directed Mutagenesis Kit; New England Biolabs, Ipswich, MA, USA) and validated via Sanger-Sequencing. Capped mRNA was produced via the mMESSAGE mMACHINE™ SP6 Transcription kit (Thermo Fisher Scientific, Waltham, MA, USA), purified and validated by RNA gel electrophoresis.

<u>Mouse methods</u>

RT-qPCR analysis of *Frmpd4* expression in mouse tissues

RNA was extracted from multiple tissues from WT male C57BL/6 (P7) mice following standard protocols (Vikhe Patil et al. 2015). RNA quality was assessed with a NanoDrop spectrophotometer (NanoDrop Technologies, Wilmington, DE). cDNA was produced using the SuperScript III First-Strand Synthesis SuperMix RT-PCR kit (Invitrogen, Karlsruhe, Germany). cDNA from different mouse tissues were generated (1 µg whole organ RNA) and analyzed by qPCR. The *Frmpd4* (NM_001033330) and *Hprt* cDNA regions of interest were by amplified using standard PCR conditions using primers mm *Frmpd4* Ex10-11 F and R and mm *Frmpd4* Ex16-17 F and R. RT-qPCR of *Frmpd4* and the endogenously expressed genes

medRxiv preprint doi: https://doi.org/10.64898/2026.03.27.26349271; this version posted March 30, 2026. The copyright holder for this preprint (which was not certified by peer review) is the author/funder, who has granted medRxiv a license to display the preprint in perpetuity.
It is made available under a CC-BY 4.0 International license .

Eef2 (NM_007909) and *Tbp* (NM_013684) were performed according to standard protocols. Primers are listed in Supplementary Table 1.

Reverse transcription was performed using 1 µg RNA per sample and FIREScript RT cDNA synthesis kit (Solis BioDyne, Tartu, Estonia) according to manufacturer's instructions. Relative expression levels were generated using qPCR HOT Fire Pol Eva Green qPCR Mix Plus (Solis BioDyne, Tartu, Estonia) according to standard protocols. Two different intron-spanning primer sets were selected for analysis of *Frmpd4* expression (targeting exons 8-9, 10-11 and 16-17) and *Eef2*, as well as *Tbp* were used for cDNA normalization. The PCR reactions were performed in technical triplicates. The qPCR reactions were performed using 364-well-plates and the Quantstudio Real-Time PCR system (Life Technologies, Darmstadt, Germany). The following program was used: 95°C 15 min, 40x [95°C, 15 s, 60°C 20 s, 72°C 20 s] followed by the generation of a melting curve using temperatures from 60°C until 95°C. Primer sequences are listed in Supplementary Table 1.

In silico analysis of _Frmpd4_ expression in the cochlea and spiral ganglion neurons (SGNs) in the WT mouse

We performed an *in silico* analysis of the expression of *Frmpd4* during mouse development using various publicly available RNA-seq datasets. First, we studied the expression of *Frmpd4* across several cochlear tissues in the WT P8 mouse using the dataset "Xenium Analysis of P8 WT Mouse Inner Ear Sections (Violin)," generated using the Xenium analyzer (10x Genomics). This dataset was generated by fixing P8 WT cochleae that were harvested, fixed in paraformaldehyde, decalcified, and embedded in paraffin wax. Tissue was sectioned in 5 µm sections and placed on Xenium slides. The standard Xenium protocol was followed and custom probes were applied. A Xenium Analyzer was used to decode the probes. We next studied SGN expression, using the Gene Expression Omnibus (GEO) Series GSE132925 (Li et al. 2020) that assessed expression in WT mice at embryonic day (E)15.5, P1, P8, P14, and P30. This dataset was generated using RNA-seq on manually dissected WT mouse SGNs at these five distinct ages. In order to identify SGN-specific genes, the transcriptomes of the SGNs were compared with those of the inner ear hair cells at P12 and glial cells at P8. Finally, analysis of *Frmpd4* expression in SGN types Ia, Ib, Ic, and 2 were analyzed using GEO Series GSE114997 (Shrestha et al. 2018). This previously generated dataset isolated cochlear sensory neurons from P25 to P27 mice (Genotypes: $Bhlhb5^{Cre}$/+; *Ai14*/+ and $Bhlhb5^{Cre}$/+; *Ai14*/+; $Vglut3^{-/-}$). Gene expression was measured using scRNA-seq. The dataset consists of gene expression from 63 type Ia, 71 type Ib, 45 type Ic and 7 type II neurons. All data were visualized in the gene expression analysis resource (gEAR) portal (Orvis et al. 2021).

Immunofluorescence in mouse experiments

medRxiv preprint doi: https://doi.org/10.64898/2026.03.27.26349271; this version posted March 30, 2026. The copyright holder for this preprint (which was not certified by peer review) is the author/funder, who has granted medRxiv a license to display the preprint in perpetuity.
It is made available under a CC-BY 4.0 International license .

For immunohistochemical analysis of cochlea, mice were deeply anesthetized with CO_2 and perfused intracardially with 4% PFA (Roth, #03353) in 1□M PBS pH 7.4 at room temperature. Cochleae were postfixed over night with 4% PFA in 1□M PBS pH 7.4 and afterwards decalcified in 125□mM EDTA (Invitrogen, #AM9262) for 48□h, while EDTA was changed after 24h. For whole mount staining, the cochlear turns were separated and covered in a 24 well plate with 1% PFA in 1□M PBS till preparations are finished. For cryosections, the mouse cochleae were incubated in ascending concentrations of sucrose (Roth, #90971) along with an additional suspension of Compound Tissue Tek (Sakura, #4583). The cochleae were afterwards embedded and frozen at -20°C in pure Compound Tissue Tek and cut in 5□µm sections. The organ of Corti and the spiral ganglia of whole mount and cryosections was first blocked and permeabilized with a solution of 10% normal horse serum (Merck, #H0146), 1% bovine serum albumin (Roth, #80762), 1% Triton X-100 (Serva, #37240), and 0.1% Tween20 (Sigma, #P1379) in 1□M PBS pH 7.4 at room temperature. Afterwards, the tissue was incubated with a primary antibody solution containing 3% normal horse serum, 1% bovine serum albumin, 0.3% Triton X-100, and 0.1% Tween20 with primary antibodies for βII-tubulin (mouse monoclonal, R&D System, #MAB1192 clone Tuj1, dilution 1:1000) and FRMPD4 (rabbit, polyclonal, dilution 1:500) (Hu et al. 2012) incubated overnight at 4°C. On the next day, primary antibodies were detected with secondary antibody Alexa488 (donkey anti-mouse IgG (H+L) Highly Cross-Adsorbed Secondary Antibody, Invitrogen, A21206), and Alexa-555 (donkey anti-rabbit IgG (H+L) Highly Cross-Adsorbed Secondary Antibody, Invitrogen, A32794 or goat anti-rabbit IgG (H+L) Cross-Adsorbed Secondary Antibody, Invitrogen, A21428; dilution 1:1000) and DAPI (dilution 1:5000; Invitrogen). Counterstaining of F-Actin was performed by fluorescent conjugated Phalloidin staining according to manufacturer instructions. Images of stained cryosections were taken with a Nikon confocal laser scanning microscope A1+ and analyzed with Fiji/ImageJ software. Images of the whole mount staining were taken with an Olympus IX81 microscope equipped with an Olympus FV1000 confocal laser scanning system, an FV1000 SPD spectral detector and diode lasers operating at 473, 559 and 651 nm. Images were acquired using an Olympus UPLSAPO 40x objective (oil, numerical aperture: 1.3). For high-resolution confocal scanning, a pinhole aperture representing a diffraction disk was used. Whole-mount images of the organ of Corti were taken at 300 nm intervals along the z-axis. The z-stacks, brightness and contrast of the images were adjusted using ImageJ for better visualization.

Auditory brainstem response (ABR) testing in mice

Hearing thresholds were measured by click and tone burst ABR (6, 8, 12, 16, and 24 kHz) for *Frmpd4*$^{-/-}$ and WT mice. ABR testing from 14 male mice included four and two *Frmpd4*$^{-/-}$ mice and six and two WT mice at 3 and 5 months of age, respectively. ABRs were measured as

medRxiv preprint doi: https://doi.org/10.64898/2026.03.27.26349271; this version posted March 30, 2026. The copyright holder for this preprint (which was not certified by peer review) is the author/funder, who has granted medRxiv a license to display the preprint in perpetuity.
It is made available under a CC-BY 4.0 International license .

previously described (Lauer and May 2011; McGuire et al. 2015; Schrode et al. 2022; Schrode et al. 2018). Mice were anesthetized with 100 mg/kg ketamine and 10 mg/kg xylazine and placed on a heating pad to maintain a temperature of 37°C. Reference, active, and ground platinum needle electrodes were placed subcutaneously behind the left pinna, at the dorsal midline of the skull, and in the left hind leg, respectively. Responses were amplified and filtered from 300 to 3000 Hz and averaged over 300 repetitions. Stimulus generation and ABR measurements were controlled and collected using computer programming modules (Tucker-Davis Technologies, Alachua, FL, USA) and custom Matlab-based software. Averaged ABR waveforms were obtained by presenting clicks or 5 ms pure-tone pips (0.5□ms $\cos^2$ onset/offset) at a rate of 20 stimuli/s presented in free field at a distance of 30 cm from the vertex of the skull. Hearing thresholds (dB SPL) were measured by presenting a descending series of stimulus levels in 5- or 10-dB steps until no discernable response was detected. Maximum ABR thresholds were capped at 108 dB SPL due to system output limits. The ABR signal magnitude was calculated from averaged peak-to-peak voltage in a 10-ms time window beginning 1 ms after stimulus onset. Threshold was determined to be the sound level at which the ABR amplitude exceeded the background noise by two standard deviations. A mixed model was used to test for significant differences in ABR thresholds between *Frmpd4*$^{-/-}$ and WT mice, including subject ID as a random effect to account for repeated measures.

medRxiv preprint doi: https://doi.org/10.64898/2026.03.27.26349271; this version posted March 30, 2026. The copyright holder for this preprint (which was not certified by peer review) is the author/funder, who has granted medRxiv a license to display the preprint in perpetuity.
It is made available under a CC-BY 4.0 International license .

Supplementary Tables

Supplementary Table 1. Primers used in the study

Primer name	Sequence (5' to 3')	Amplicon length	Purpose
**Human**			
FRMPD4 c.3755 F	CAAGGCTCTTCAGTGGATG	332 bp	Genotyping
FRMPD4 c.3755 R	GCTGTACCTGTGGTTTCCT		
**Zebrafish**			
zf actin F	GAGAAGATCTGGCATCACACCTTC	607 bp	zf RT-PCR control
zf actin R	GGTCTGTGGATACCGCAAGATTC		
zf frmpd4_2 riboprobe F	ACGCTGCTGCGTTTGATTAT	460 bp	zf in situ riboprobe synthesis
zf frmpd4_2 riboprobe R	TTGGTGTTGATCACATGACTGA		
frmpd4 sgRNA f3_1	TAGGCGCAGAGTCAGGTCGAGA	NA	zf CRISPR guide f3, Cloning in pDR274
frmpd4 sgRNA f3_2	AAACTCTCGACCTGACTCTGCG		
frmpd4 sgRNA r2_1	TAGGCATCAATGGACGAACACT	NA	zf CRISPR guide r2, Cloning in pDR274
frmpd4 sgRNA r2_2	AAACAGTGTTCGTCCATTGATG		
zf_frmpd4_gDNA_fwd_f3r2	CCTTTCAGGGAAGCAATCAA	662 bp	zf CRISPR genotyping
zf_frmpd4_gDNA-rev_f3r2	ACCCCAAGGTCATGGGTTAT		
zf frmpd4 Ex11gDNA2 F	TGTGTGTTTTTCTCATGGTC	375 bp	zf sa12377 genotyping
zf frmpd4 Ex11gDNA2 R	GTTCAGAAATGTGTATCTCTCC		
zf_frmpd4_Ex14_fwd	ACGAGGAGGACAGTGACGAT	428 bp (cDNA) 758 bp (pre-mRNA)	zf RT-PCR primer for control of Splice Morpholino function
zf_frmpd4_Ex15_rev	GTACCCCTCTGCTGTGGCTA		
ef1a1_f01	GCCCCTGGACACAGAGACTTCATCA	211 bp (cDNA)	zf RT-PCR control primer
ef1a1_r01	AAGGGGGCTCGGTGGAGTCCAT		
**Mouse**			
mm cDNA Hprt F	CAGGACTGAAAGACTTGCTC	402 bp	Mouse RT-PCR
mm cDNA Hprt F	AAGTCTGGCCTGTATCCAAC		
mm Frmpd4 cDNA F	ATTCAGCCCTATCCATCTCC	588 bp	Mouse RT-PCR
mm Frmpd4 cDNA R	CTGTTTGGTGGTGACAGTAGC		
mm Eef2 RT-qPCR F	GCCATGGGTATTAAGAGCTGC	89 bp	Mouse RT-qPCR
mm Eef2 RT-qPCR R	TAGAAGCGGCCTTTGTCAGA		
mm Tbp RT-qPCR F	ACCGTGAATCTTGGCTGTAAAC	86 bp	Mouse RT-qPCR
mm Tbp RT-qPCR R	GCAGCAAATCGCTTGGGATTA		
mm Frmpd4 Ex 8-9 F	AGCCCTATCCATCTCCCAAA	81 bp	Mouse RT-qPCR
mm Frmpd4 Ex 8-9 R	AGCGCACTTTGACTGGATTG		
mm Frmpd4 Ex 10-11 F	CACCTCCATCAAGGATGTCA	78 bp	Mouse RT-qPCR
mm Frmpd4 Ex 10-11 R	AGCATGAGGGAGAAGTGTTCA		
mm Frmpd4 Ex 16-17 F	GCTAGATGTGAAGCCCATTACAC	90 bp	Mouse RT-qPCR
mm Frmpd4 Ex 16-17 R	ACAAGCAGCCGGTAGTATCCA		
Drosophila			
ngh-gRNA1 Fw	CTTGTTTATTGCAGCTTATAATGG	-	*guide-RNA1 (downstream)*
ngh-gRNA1 Rv	GAGCAGAGAAATGTTGCAC	-	
ngh-gRNA2 Fw	TTCAGATGAAAGCCTTAGCT	-	*guide-RNA (upstream)*
ngh-gRNA2 Rv	TCATGTCAAGGTCTTCTCG	-	
α-tubulin RT-qPCR F	TGTCGCGTGTGAAACACTTC	96 bp	*Drosophila* RT-qPCR
α-tubulin RT-qPCR R	AGCAGGCGTTTCCAATCTG		
EF1 RT-qPCR F	GCGTGGGTTTGTGATCAGT	125 bp	*Drosophila* RT-qPCR
EF1 RT-qPCR R	GATCTTCTCCTTGCCCATCC		
CG42788 Ex2-3 F	CTTGGAGGCCATACAGGAAA	185 bp	*Drosophila* RT-qPCR
CG42788 Ex2-3 R	AGGAACGACCCTTGGAGTCT		
CG42788 Ex5-6 F	ATGGCCAATGTGCTGAAGGT	173 bp	*Drosophila* RT-qPCR
CG42788 Ex5-6 R	TTGTTGCGCTTGAGGCTCTT		
CG42788 Ex8-9 F	ACAAACCAAAGAGAGGGCGT	116 bp	*Drosophila* RT-qPCR

medRxiv preprint doi: https://doi.org/10.64898/2026.03.27.26349271; this version posted March 30, 2026. The copyright holder for this preprint (which was not certified by peer review) is the author/funder, who has granted medRxiv a license to display the preprint in perpetuity.
It is made available under a CC-BY 4.0 International license .

CG42788 Ex8-9 R	AAGTCCTCGATGGCGGAAAT		
CG42788 Ex2 F	CCCATCTGGATGTACGGAAGT	480 bp	*Drosophila* 607756 genotyping
CG42788 MiMIC 1 R	CGAATTAATAGTGGTTGGGGC		
CG42788 MiMIC 2 F	GTGTTAAACATTGCGCACTGC	455 bp	*Drosophila* 607756 genotyping
CG42788 Ex4 R	TGGCACACCAGGAGATTTACC		
**Cloning**			
hu_frmpd4_fl_F_BamHI	tcgaGGATCCATGGATGTCTTCAG CTTTGTGA	3669 bp human full-length cDNA cloning	
hu_frmpd4_fl_R_XhoI	tcgaCTCGAGCTACACTGTGGTTT CCTTAATCTT		
hu_frmpd4_sequ_primer_rev1	TCACTTCCGAGCGCTTTTCT	1377-1396	Sequencing
hu_frmpd4_sequ_primer_fwd1	AGAAAAGCGCTCGGAAGTGA	1396-1377	
hu_frmpd4_sequ_primer_rev2	TGCGGGAGAGGTTTTCTGAC	2868-2887	
hu_frmpd4_sequ_primer_fwd2	ACCTCTCCCGCATGTTCTTG	2887-2868	
hu_frmpd4_sequ_primer_fwd3	CTTCGTCCCAAAAGATCCAA	978-967	
hu_frmpd4_sequ_primer_rev3	TTTTCCCTGTTGCTGCTTCT	3405-3424	
hu_frmpd4_Ser1252Phe_Mut1_F	CCTGACGCTTtTGGGAAAGGC	Mutagenesis, Ser1252Phe	
hu_frmpd4_Ser1252Phe_Mut1_R	AATCAAGTGCTTCCCCAG		
hu_frmpd4_Ala809Thr_Mut2_F	GGCCATTGCCaCACCCCCACC	Mutagenesis, Ala809Thr	
hu_frmpd4_Ala809Thr_Mut2_R	ATGTTCAAGGAACGCAACAGGAA G		

Abbreviation: NA, not applicable; zf, zebrafish

medRxiv preprint doi: https://doi.org/10.64898/2026.03.27.26349271; this version posted March 30, 2026. The copyright holder for this preprint (which was not certified by peer review) is the author/funder, who has granted medRxiv a license to display the preprint in perpetuity.
It is made available under a CC-BY 4.0 International license .

Supplementary Table 2. Exome filtering details for Family 1

Total combined familial transcript variants	304,240
Exonic transcript variants	11,846
Transcript variants with Qscore ≥ 30	9,508
Transcript variants with a MAF ≤ 0.01 (1000 Genomes Project and Exome Variant Server)	4,703
In house MAF ≤ 0.01 (n = 511 exomes)	1,819
Exclude transcript variants in artifact-prone genes*	1,758
Total missense, stop gain, stop loss, start gain, start loss, splice, indel, total count transcript variants	1,327
Total synonymous transcript variants	401
Transcript-independent variants: Missense, stop gain, stop loss, start gain, start loss, splice, indel, total count	714
Pedigree-filtering variant analysis	
Autosomal recessive mode of inheritance:	
Homozygous in III:2 and III:3	0
Heterozygous in II:2 and II:3 and not inherited from a parent	0
Compound heterozygous in III:2 and III:3	0
Autosomal dominant mode of inheritance:	
Heterozygous in III:2, III:3, II:3, absent in III:2, coverage ≥ 20x, MAF ≤ 0.00002[a,b]	14
Heterozygous/Hemizygous variants predicted pathogenic by 2/3 of *in silico* tools	3

**HLA*s, *MAGE*s, *MUC*s, *NBPF*s, *OR*s, *PRAME*s
[a]Adhering to guidelines for variant filtering from Oza et al., 2018.
[b]Shown in Supplementary Table 3.

Supplementary Table 3. Heterozygous variants identified in the affected individuals in Family 1 that were inherited from a healthy parent

Gene	MIM	g. position	Ref	Alt	cDNA change	Amino acid change	rsID	gnomAD European (non-Finnish)	gnomAD all MAF	CADD	Mutation-Taster	PolyPhen-2	SIFT
BDP1[a]	607012	chr5:g.70800557	A	G	NM_018429.2:c.2351A>G	p.Lys784Arg	rs771158737	1.56e-5	3.10e-6	3.23	B 1|99	B 0.001	T 0.60
COA1	614769	chr7:g.43684932	T	C	NM_018224.3:c.182A>G	p.Gln61Arg	rs2539257437	0	0	15.01	B 7|93	B 0.255	D 0.03
E2F8	612047	chr11:g.19251845	G	A	NM_024680.3:c.1301C>T	p.Pro434Leu	rs1390528068	9.32e-6	6.82e-6	22.10	B 5|95	B 0.418	D 0.04
FAAP100	611301	chr17:g.79514704	T	-	NM_025161.5:c.1404delA	p.Val469Cysfs*4	--	0	0	--	--	--	--
HINT3	609998	chr6:g.126278146	G	C	NM_138571.4: c.23G>C	p.Arg8Pro	rs571982659	6.10e-6	4.15e-4	0.23	B 4|96	B 0.000	T 0.21
MYBPH	160795	chr1:g.203139523	A	C	NM_004997.2:c.989T>G	p.Leu330Arg	rs376467645	2.20e-5	1.67e-5	27.50	D 59|41	PrrD 1.00	D 0.00
MYOZ3	610735	chr5:g.150051402	G	A	NM_133371.4:c.356G>A	p.Gly119Asp	rs1231422914	0	0	4.02	B 1|99	B 0.117	T 0.36
NKPD1	--	chr19:g.45656477	G	T	NM_198478.3:c.1218C>A	p.Ser406Arg	--	0	0	29.00	B 43|57	--	D 0.01
NME4	601818	chr16:g.450325	A	G	NM_005009.2:c.547A>G	p.Ser183Gly	rs779063691	9.34e-6	6.87e-6	11.27	B 7|93	PoD 0.65	T 0.33
PLIN3	602702	chr19:g.4844709	C	T	NM_005817.4:c.931G>A	p.Gly311Ser	rs2030025374	0	0	17.57	B 8|92	PoD 0.638	T 0.17
SVEP1	611691	chr9:g.113173770	T	C	NM_153366.3:c.6221A>G	p.Asp2074Gly	--	0	0	17.44	B 12|88	B 0.000	T 0.28
TAPBPL	607081	chr12:g.6562260	G	A	NM_018009.4:c.92G>A	p.Arg31Gln	rs146769470	2.50e-5	1.30e-1	16.41	B 1|99	B 0.231	T 0.42
TRIM16	609505	chr17:g.15532464	C	A	NM_006470.3:c.1160G>T	p.Arg387Leu	rs2549715759	8.48e-7	6.20e-7	26.00	B 36|64	PrD 1.00	T 0.22
ZNHIT2	604575	chr11:g.64884074	T	A	NM_014205.2:c.1052A>T	p.Glu351Val	rs2539168289	8.50e-7	6.21e-7	25.4	B 17|83	PrD 0.998	T 0.12

[a]*BDP1* is associated with autosomal dominant non-syndromic hearing loss (Girotto et al. 2013). The Deafness Variation Database prioritizes this variant as likely benign. Genomic coordinates are in GRCh37/hg19. CADD ≥20 was considered deleterious.

Abbreviation: B, benign; D damaging; DC, disease causing; PrD, probably damaging; PoD, possibly damaging, PrD, probably damaging; T, tolerated

medRxiv preprint doi: https://doi.org/10.64898/2026.03.27.26349271; this version posted March 30, 2026. The copyright holder for this preprint (which was not certified by peer review) is the author/funder, who has granted medRxiv a license to display the preprint in perpetuity.
It is made available under a CC-BY 4.0 International license .

Supplementary Table 4. Variants in known hearing loss genes and candidate genes identified in affected individuals through exome sequencing

Chr	bp (hg19)	Ref	Alt	Zyg	Gene	cDNA change	aa change	rs	gnomAD MAF	CADD	MT	PolyPhen-2	SIFT
Family 1 pedigree filtering													
11	17,531,325	G	A	Het	*USH1C*	NM_153676.3:c.1591C>T	p.Arg531Cys	rs140528164	0.00007379	28.3	DC	PD	T
X	12,736,700	C	T	Hemi	*FRMPD4*	NM_014728.3:c.3755C>T	p.Ser1252Phe	NR	NR	25.4	DC	PD	D
X	18,972,407	C	T	Hemi	*PHKA2*	NM_000292.2:c.202G>A	p.Asp68Asn	rs866514095	0.00002495	25.1	DC	PD	D
Family 2, individual IV:1													
1	110,019,548	C	CTA	Hom	*SYPL2*	NM_001040709.1:c.406_407dup	p.Leu137fsThrfs*21	NR	NR	NR	DC	NR	NR
10	73,570,263	C	T	Het	*CDH23*	NM_022124.5:c.9014C>T	p.Ala3005Val	rs188966938	0.00001605	22.5	DC	B	D
12	112,326,421	G	A	Hom	*MAPKAPK5*	NM_139078.2:c.1099G>A	p.Gly367Ser	NR	NR	34	DC	DC	D
17	76,046,996	G	T	Hom	*TNRC6C*	NM_001142640.2:c.2483G>T	p.Gly828Val	NR	NR	23.6	DC	NR	D
X	127,350,003	G	A	Hemi	*FRMPD4*	NM_014728.3:c.2425G>A	p.Ala809Thr	NR	0.000005600	27.9	DC	PD	T

Abbreviations: aa, amino acid; alt, alternate base; bp, base pair position (GRCh37, hg19); CADD, Combined Annotation Dependent Depletion; Chr, chromosome; D, deleterious; DC, disease causing; gnomAD MAF, genome aggregation database minor allele frequency; NR, not reported; ref, reference; T, tolerated; zyg, zygosity

medRxiv preprint doi: https://doi.org/10.64898/2026.03.27.26349271; this version posted March 30, 2026. The copyright holder for this preprint (which was not certified by peer review) is the author/funder, who has granted medRxiv a license to display the preprint in perpetuity.
It is made available under a CC-BY 4.0 International license .

medRxiv preprint doi: https://doi.org/10.64898/2026.03.27.26349271; this version posted March 30, 2026. The copyright holder for this preprint (which was not certified by peer review) is the author/funder, who has granted medRxiv a license to display the preprint in perpetuity.
It is made available under a CC-BY 4.0 International license .

Supplementary Table 5. Exome filtering details for Family 2, individual IV:1

Total variants	99,819
Exonic transcript variants	25,845
Transcript variants with Qscore ≥ 30	15,043
Transcript variants with a MAF ≤ 0.00007 (1000 Genomes Project and Exome Variant Server)	869
Exclude transcript variants in artifact-prone genes*	507
Total missense, stop gain, stop loss, start gain, start loss, splice, indel	348
Total synonymous variants	159
Autosomal recessive mode of inheritance predicted to be pathogenic by 2/3 of *in silico* tools:	
Homozygous variants	4
Compound heterozygous variants	0
X-linked mode of inheritance pathogenic by 2/3 of *in silico* tools:	
Hemizygous variants	1

Supplementary Table 6. Homozygous variants identified in individual IV:1 from Family 2

Gene	MIM	g. position	Ref	Alt	cDNA change	Amino acid change	rsID	gnomAD all ME	gnomAD all MAF	CADD score, scaled	Mutation-Taster	PolyPhen-2	SIFT
DPP4	102720	chr2:g.162,903,933	T	C	NM_001935.3:c.173A>G	p.Tyr58Cys	rs756475707	0	2.54e-6	23.60	B 14|86	PrD 1.0	T 0.13
MAPKAPK5	606723	chr12:g.112,326,421	G	A	NM_139078.2:c.1099G>A	p.Gly367Ser	--	0	0	23.90	D 58|42	PrD 1.0	D 0.00
PRDM9	609760	chr5:g.23,522,762	C	A	NM_020227.2:c.650C>A	p.Ala217Asp	--	0	0	22.00	B 13|87	PrD 0.983	T 0.08
SYPL2	--	chr1: g.110,019,548	C	CTA	NM_001040709.1: c.406_407dupTA	p.Leu137Thrfs *21	--	--	--	--	--	--	--
TNRC6C	610741	chr17:g.76,046,996	G	T	NM_001142640.1:c.1853G >T	p.Gly618Val	--	--	--	23.60	B 21|79	--	D 0.00

Abbreviations: B, benign; D damaging; DC, disease causing; ME, Middle Eastern; PrD, probably damaging; PoD, possibly damaging, PrD, probably damaging; T, tolerated

medRxiv preprint doi: https://doi.org/10.64898/2026.03.27.26349271; this version posted March 30, 2026. The copyright holder for this preprint (which was not certified by peer review) is the author/funder, who has granted medRxiv a license to display the preprint in perpetuity.
It is made available under a CC-BY 4.0 International license .

Supplementary Table 7. *FRMPD4* variants with clinical summary

***FRMPD4* variant**	**Inheritance**	**Phenotype**	**Reference**
~632 kb duplication including part of *FRMPD4* (Exon 1 duplicated)	Maternal	Mild ID and autism	(Honda et al. 2010)
~66 kb microdeletion including exon 2	Maternal	Delayed gross motor development, delayed speech and language development, severe to profound ID, autism, hypotonia, spasticity, brisk deep tendon reflexes, ataxia, reduced occipitofrontal circumference, epilepsy, strabismus	(Piard et al. 2018)
c.380C>T, p.Pro127Leu (Exon 4)	Maternal	ID	(Trujillano et al. 2017)
c.856C>T, p.Arg286* (Exon 9)	Maternal	Delayed gross motor development, delayed speech and language development, moderate ID, hyperactivity (aggressiveness/autism)	(Piard et al. 2018)
c.1657T>C, p.Cys553Arg (Exon 15)[1]	*de novo*	Mild to moderate ID, developmental delay, mild facial features, absent speech, autism spectrum disorder	(Hu et al. 2016)
c.1657T>C, p.Cys553Arg (Exon 15)[1]	*de novo*	Delayed gross motor development, delayed speech and language development, severe ID, autism, strabismus	(Piard et al. 2018)
c.1772A>C, (p.Glu591Ala) (Exon 15)	Maternal	Severe ID and language impairment	(Pan et al. 2024)
c.1852delT, p.Cys618Valfs*8 (Exon 15)	Maternal	Mild to severe ID with variable seizures, lack of speech/poor speech, behavioural problems	(Hu et al. 2016)
c.2425G>A, p.Ala809Thr (Exon 15)	Maternal	Non-syndromic sensorineural hearing loss	Present study
c.2641G>A, p.Val881Met (Exon 15)	Maternal	Isolated epilepsy	(Li et al. 2024)
c.2800C>T, p.Arg934Cys (Exon 16)	Maternal	Isolated epilepsy	(Li et al. 2024)
c.2816C>T, p.Thr939Ile (Exon 16)	Maternal	Epilepsy with ID	(Li et al. 2024)
c.3061T>C, p.Tyr1021His (Exon 16)	Maternal	Isolated epilepsy	(Li et al. 2024)
c.3623T>C, p.Phe1208Ser (Exon 16)	Maternal	Isolated epilepsy	(Li et al. 2024)
c.3755C>T, p.Ser1252Phe (Exon 16)	Maternal	Non-syndromic sensorineural hearing loss	Present study

FRMPD4 (NM_001368397.1) has 17 exons; [1] Same *de novo* variant seen in two patients

Abbreviation: ID, intellectual disability

medRxiv preprint doi: https://doi.org/10.64898/2026.03.27.26349271; this version posted March 30, 2026. The copyright holder for this preprint (which was not certified by peer review) is the author/funder, who has granted medRxiv a license to display the preprint in perpetuity. It is made available under a CC-BY 4.0 International license .

medRxiv preprint doi: https://doi.org/10.64898/2026.03.27.26349271; this version posted March 30, 2026. The copyright holder for this preprint (which was not certified by peer review) is the author/funder, who has granted medRxiv a license to display the preprint in perpetuity.
It is made available under a CC-BY 4.0 International license .

Supplementary Figures

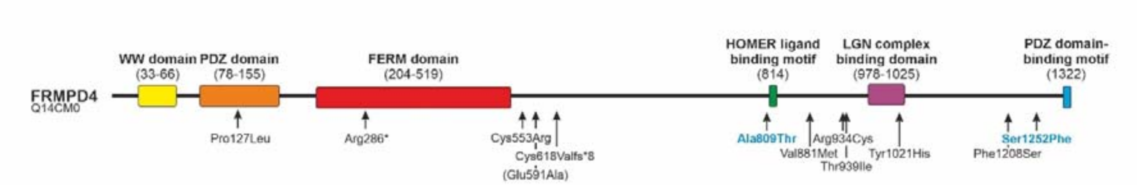

Supplementary Figure 1. Schematic overview of *FRMPD4* protein domain structure and disiease associated genetic variants reported to date
Variants exclude (Honda et al. 2010; Piard et al. 2018).
Numbers in brackets indicate amino acid positions in human protein (UniProt ID: Q14CM0). WW domain: rsp5-domain, WWP repeating motif (PFAM: PF00397; InterPro: IPR001202); PDZ domain (PFAM: PF00595; InterPro: IPR001478), FERM domain (PFAM: PF09379; InterPro: IPR000299), Homer ligand binding motif (PPPGFRD; InterPro: IPR019588), LGN complex binding domain (InterPro: IPR031938), PDZ domain-binding motif (InterPro: IPR001478) (Lee et al. 2008; Takayanagi et al. 2015).

medRxiv preprint doi: https://doi.org/10.64898/2026.03.27.26349271; this version posted March 30, 2026. The copyright holder for this preprint (which was not certified by peer review) is the author/funder, who has granted medRxiv a license to display the preprint in perpetuity.
It is made available under a CC-BY 4.0 International license .

A Alignment

species	ENSEMBL ID	protein length [aa]	overall protein aa identity to human [%]	PDZ domain aa identity to human [%]	FERM domain aa identity to human [%]	UniProt ID
human	ENSP00000370057	1322	100	100	100	Q14CM0
mouse	ENSMUSP00000107775	1312	90,6	98,7	99,3	A2AFR3
zebrafish	ENSDARP00000102225	1121	53,9	89,7	85,4	F1QXP3
Drosophila	FBgn0261859 / CG42788	1560	24,6	40,6	58,9	Q9VFD3

B Synteny

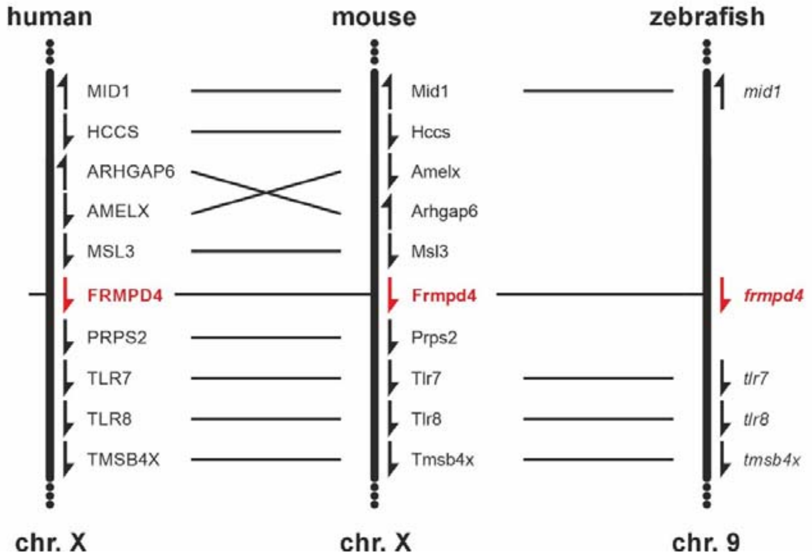

Supplementary Figure 2. Evolutionary conservation of Frmpd4 orthologues.
(A) Amino acid alignment of human, mouse, zebrafish and drosophila frmpd4 orthologous.
(B) Analysis of gene locus synteny in the human, mouse and zebrafish genome.

medRxiv preprint doi: https://doi.org/10.64898/2026.03.27.26349271; this version posted March 30, 2026. The copyright holder for this preprint (which was not certified by peer review) is the author/funder, who has granted medRxiv a license to display the preprint in perpetuity.
It is made available under a CC-BY 4.0 International license .

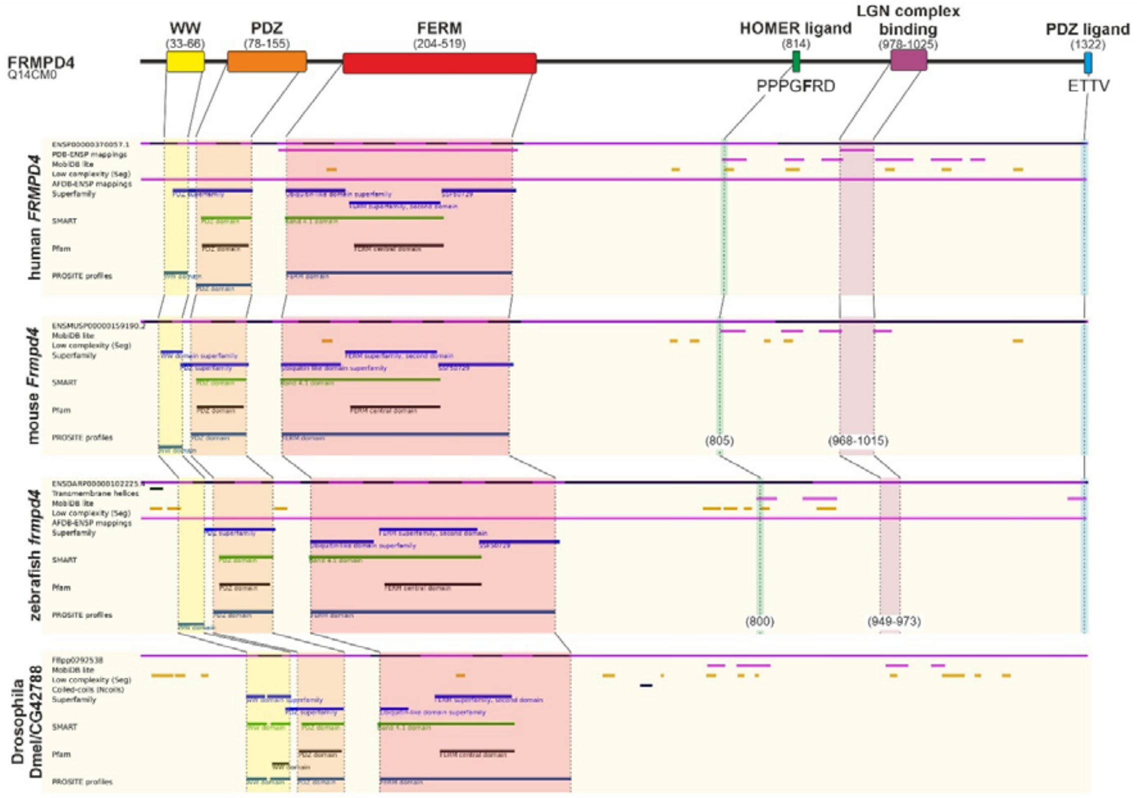

Supplementary Figure 3. Summary of FRMPD4 protein domains and domain conservation in orthologous.

Visualization of FRMPD4 protein domains. Given numbers are amino acid positions in human FRMPD4 (Uniprot ID: Q14CM0). Ensembl protein tracks and predicted domain structures of mouse, zebrafish, and *Drosophila* orthologues are aligned to human FRMPD4. Different colorations imply conserved domains.

medRxiv preprint doi: https://doi.org/10.64898/2026.03.27.26349271; this version posted March 30, 2026. The copyright holder for this preprint (which was not certified by peer review) is the author/funder, who has granted medRxiv a license to display the preprint in perpetuity.
It is made available under a CC-BY 4.0 International license .

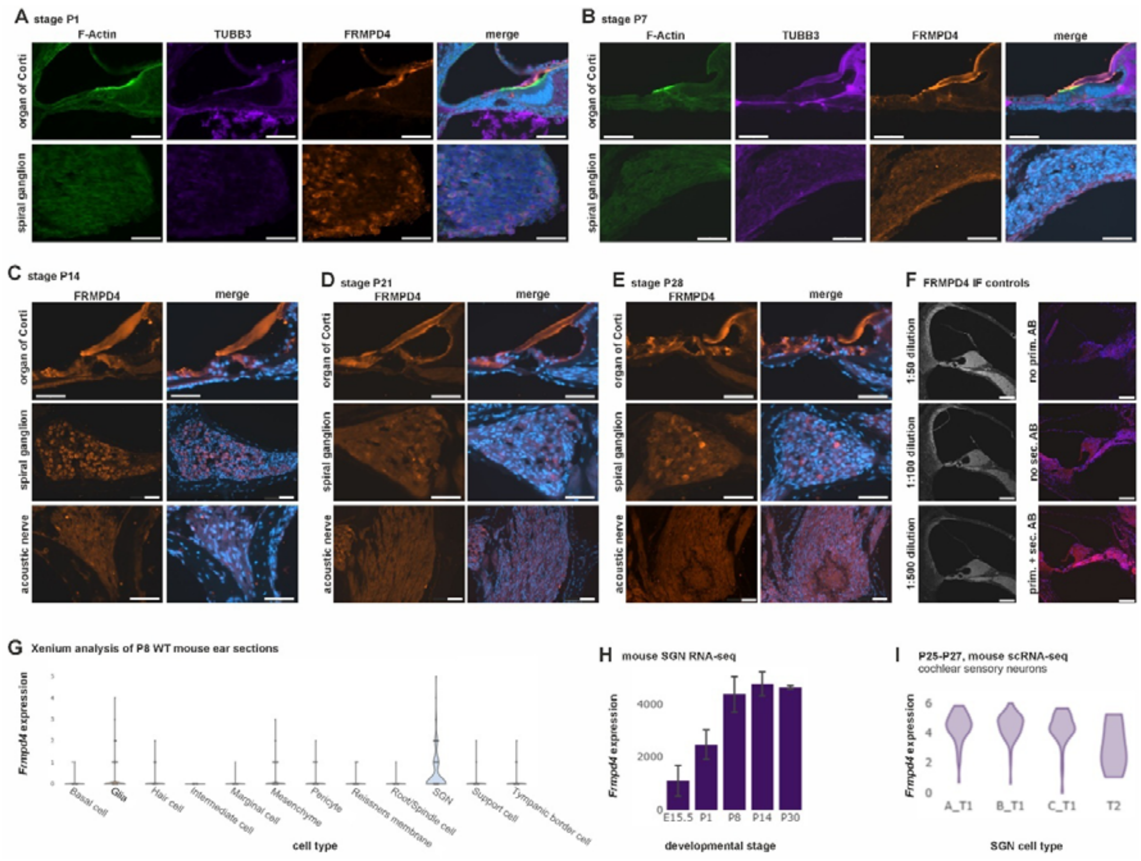

Supplementary Figure 4. Additional mouse expression data.

IF on different mouse stages and controls. Localization of FRMPD4, F-Actin and TUBB3 visualized by immunofluorescence in the organ of Corti, in the spiral ganglion and in the acoustic nerve show of mice neonates (stages **(A)**: P1; **(B)**: P7; **(C)**: P14; **(D)**: P21; **(E)**: P28). **(F)** FRMPD4 IF antibody dilution series and IF controls (no primary, no secondary, with primary and secondary antibody; similar fluorescence settings; FRMPD4 is visualized in red, nuclear counterstaining with DAPI). 5 µm sections. Scale bars = 50µm. **(G)** Violin plots of gene expression from 10x genomics xenium analysis shows highest expression in the spiral ganglion neurons (SGNs) in the P8 wild type mouse cochlea. **(H)** The expression of *Frmpd4* in the mouse SGN increases between E15.5 to P8 and then remains stable. Data were previously generated (Li et al. 2020). **(I)** Single-cell RNA sequencing shows expression of *Frmpd4* in all SGN sub-types. Data were generated previously (Shrestha et al. 2018). All data were visualized in the gene expression analysis resource (gEAR) portal (Orvis et al. 2021).

medRxiv preprint doi: https://doi.org/10.64898/2026.03.27.26349271; this version posted March 30, 2026. The copyright holder for this preprint (which was not certified by peer review) is the author/funder, who has granted medRxiv a license to display the preprint in perpetuity.
It is made available under a CC-BY 4.0 International license .

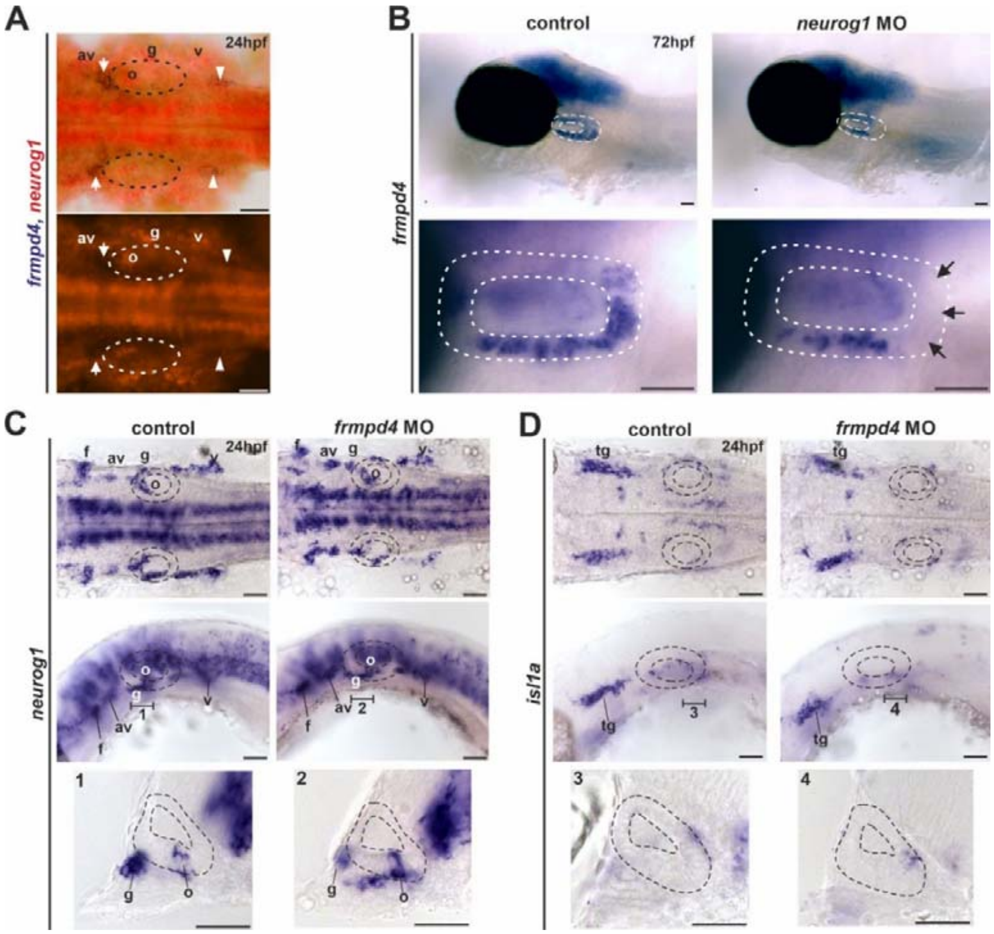

Supplementary Figure 5. *frmpd4* expression partly localizes with *neurog1* in the otic vesicle and is regulated by *neurog1.*

(A) Expression of *frmpd4* (stained blue) and *neurog1* (stained red) co-localize in the otic vesicle of 24hpf embryos (white arrows), but not in posterior lateral line precursors (white arrowheads). **(B)** Morpholino knockdown of *neurog1* results reduction of *frmpd4* expression exclusively in the posterior otic vesicle (black arrows; MO concentration: 0.25 mM; changed *frmpd4* expression pattern in control group: 0/12; changed *frmpd4* expression pattern in *neurog1* MO group: 12/28). Expression of *neurog1* **(C)** and *isl1* **(D)** in the otic vesicle is not influenced, by *frmpd4* knockdown (MO concentration: 0.5mM; investigated embryos per group and gene: >5). The otic vesicle is outlined with dashed lines. av - anteroventral lateral line placode; f - facial epibranchial placode; g -glossopharyngeal epibranchial placode; o - octaval/statoacoustic ganglion precursors; tg - trigeminal ganglion; v - vagal epibranchial placode. Scale bars indicate 50µm.

medRxiv preprint doi: https://doi.org/10.64898/2026.03.27.26349271; this version posted March 30, 2026. The copyright holder for this preprint (which was not certified by peer review) is the author/funder, who has granted medRxiv a license to display the preprint in perpetuity.
It is made available under a CC-BY 4.0 International license .

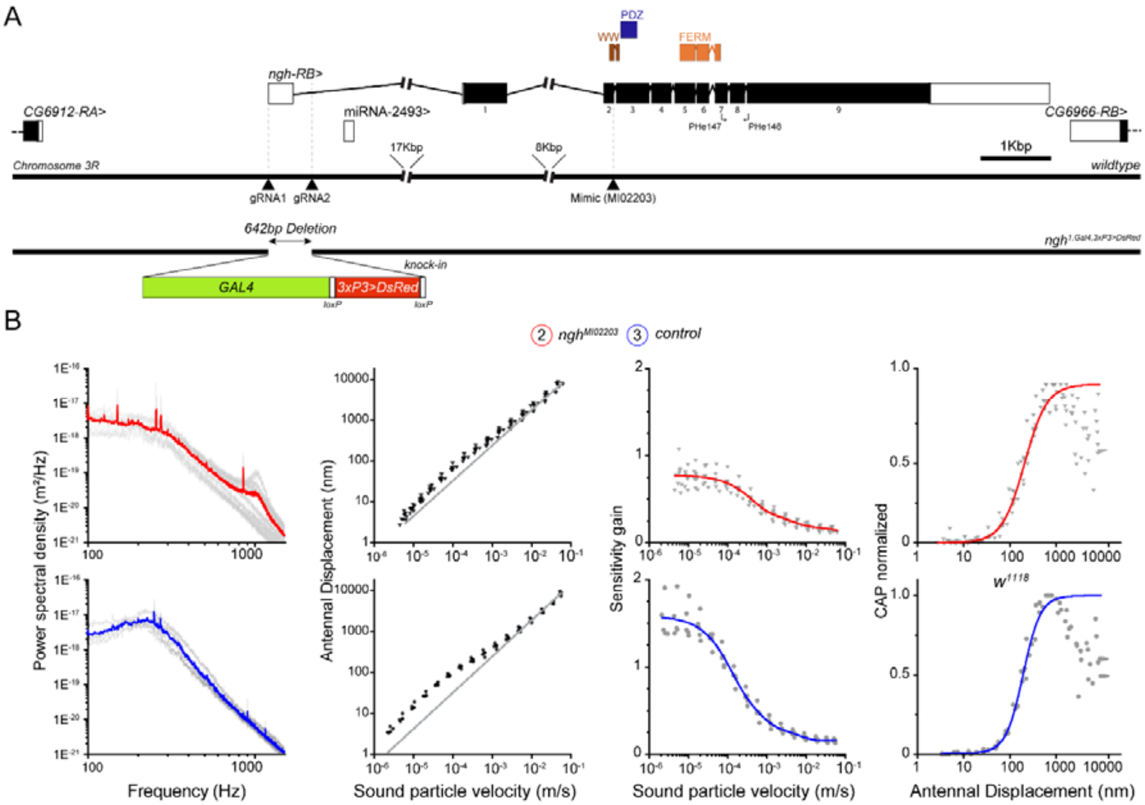

Supplementary Figure 6. Overview of the *Drosophila* CG42788-RB gene locus and sound perception quantification.

(A) Scheme of the genetic locus of CG42788-RB, the *FRMPD4* orthologue in *Drosophila*. Coding and non-coding exons are marked in black and white boxes; functional domains are shown with colored boxes. Gal4-3xP3-DsRed knock-in integration site and corresponding gRNA sites (spanning a 642bp deletion, including exon 1 and parts of intron 1/2) are shown at the bottom. **(B)** Quantification of Power spectral density to Frequency, Antennal Displacement to Sound particle velocity, Sensitivity gain to Sound particle velocity and CAP normalized to Antennal Displacement in *ngh (nicht gut hörend, marked in red)* and control (marked in blue) flies.

medRxiv preprint doi: https://doi.org/10.64898/2026.03.27.26349271; this version posted March 30, 2026. The copyright holder for this preprint (which was not certified by peer review) is the author/funder, who has granted medRxiv a license to display the preprint in perpetuity.
It is made available under a CC-BY 4.0 International license .

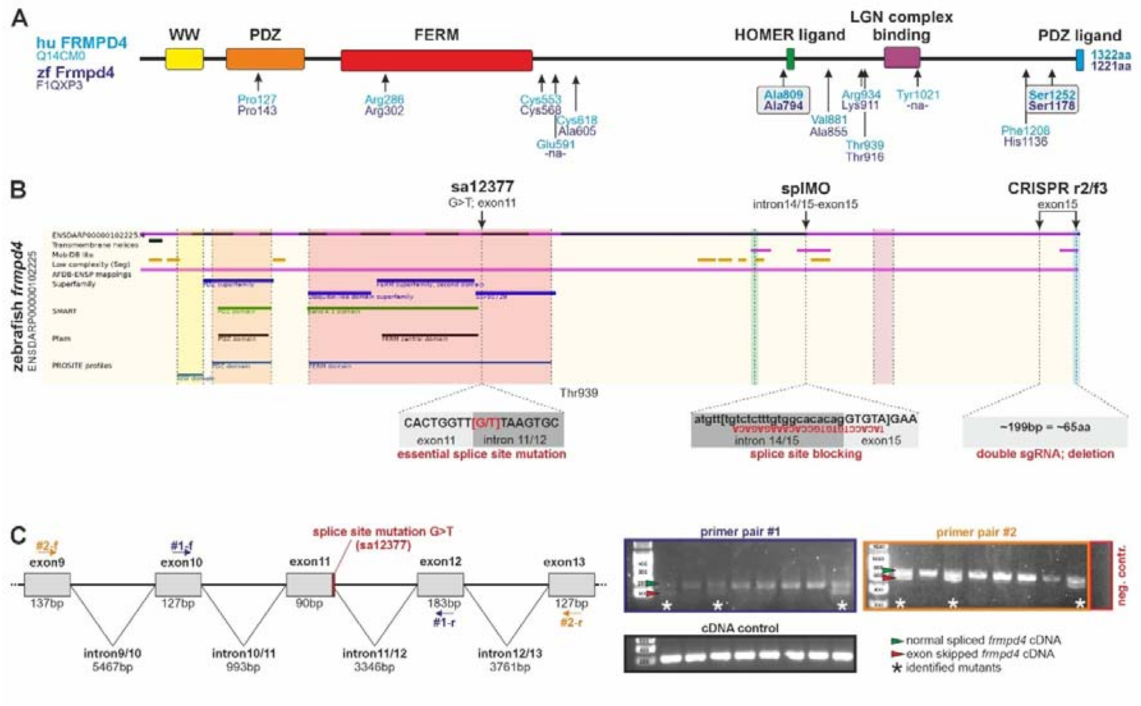

Supplementary Figure 7. Summary of Frmpd4 domains in zebrafish.

(A) Conservation of human variants in zebrafish. **(B)** Overview about used zebrafish knockdown reagents. **(C)** *frmpd4* locus overview and example of *frmpd4*^{sa12377} mutant identification by RT-PCR on cDNA from single embryos with two independent primer pairs spanning mutation site in exon11. Identified mutants (marked by asterisks) show an exon skipped PCR product (red arrowhead) in addition to a normal spliced product (green arrowhead). Amplification of *ef1a1* from the same cDNA was used as positive control. A PCR reaction without cDNA depicts a negative control.

medRxiv preprint doi: https://doi.org/10.64898/2026.03.27.26349271; this version posted March 30, 2026. The copyright holder for this preprint (which was not certified by peer review) is the author/funder, who has granted medRxiv a license to display the preprint in perpetuity.
It is made available under a CC-BY 4.0 International license .

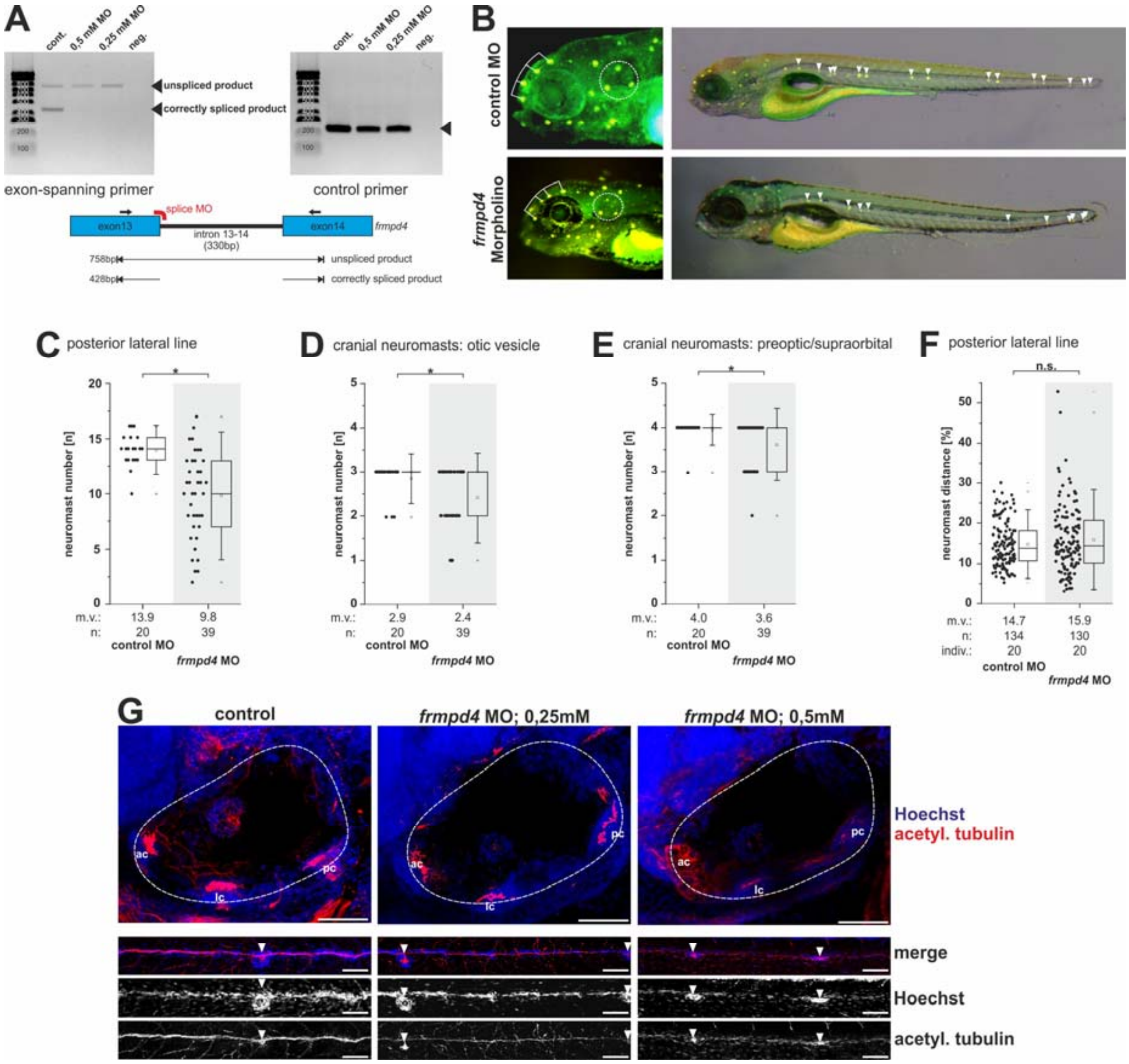

Supplementary Figure 8. Loss of function of *frmpd4* in zebrafish results in neuromast alterations in the otic vesicle and in the posterior lateral line.

(A) Functional test of *frmpd4* splice blocking Morpholino via RT-PCR (per condition 10 pooled embryos). Exon-spanning PCR primers indicate the loss of a correctly spliced *frmpd4* band after Morpholino injection, and gain of a larger, unspliced additional PCR product. PCR amplification of *ef1a1* were used as internal cDNA control. **(B)** DASPEI staining for neuromasts in *frmpd4* Morpholino injected knockdown embryos. Quantification of neuromast number in the posterior lateral line **(C)**, the otic vesicle **(D)** and the preoptical/supraorbital **(E)** indicated reduction after *frmpd4* knockdown (0.25mM Morpholino concentration, age: 4 dpf, comparison to embryos injected with standard MO). **(F)** Measurement of neuromast distance in the posterior lateral line indicated no significant change after *frmpd4* Morpholino knockdown. **(G)** Staining for acetyl tubulin after *frmpd4* Morpholino knockdown in 4 dpf larvae indicated similar to in *frmpd4*^{sa12377} mutants loss of neuronal cells and axonal projections in ventral sensory patches of the otic vesicle and interference with correct neuromast formation in the posterior lateral line. Analyzed embryos: 2 wild type controls; 4 *frmpd4* MO 0.25mM; 2 *frmpd4* MO 0.5mM.

medRxiv preprint doi: https://doi.org/10.64898/2026.03.27.26349271; this version posted March 30, 2026. The copyright holder for this preprint (which was not certified by peer review) is the author/funder, who has granted medRxiv a license to display the preprint in perpetuity.
It is made available under a CC-BY 4.0 International license .

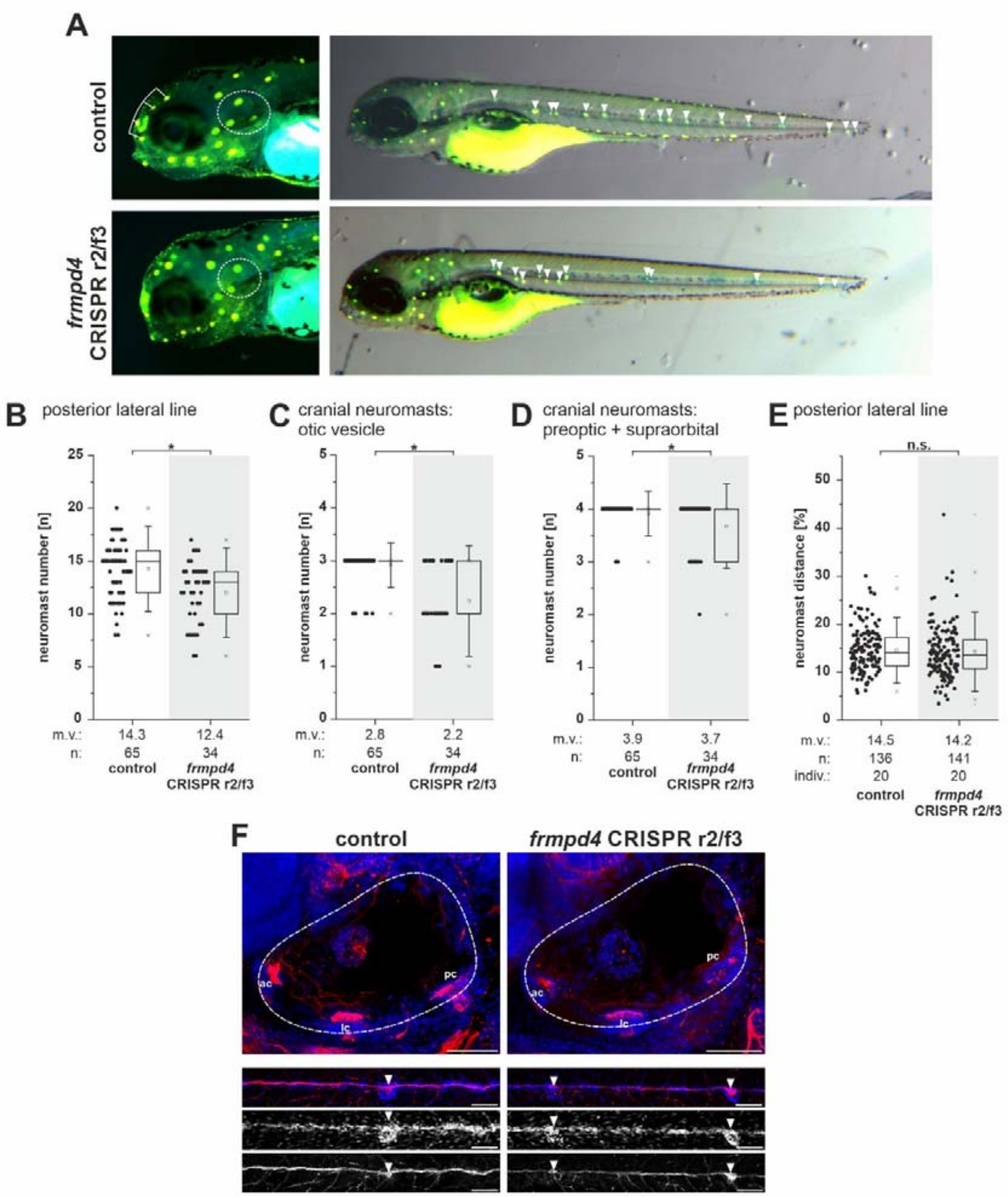

Supplementary Figure 9. *frmpd4* CRISPant in zebrafish show only mild neuromast alterations in the otic vesicle and in the posterior lateral line.

(A) DASPEI staining for neuromasts in CRISPants embryos (F0 generation, *frmpd4* sgRNA r2 and f3 injected). Quantification of neuromast number in the posterior lateral line **(B)**, the otic vesicle **(C)**, and the preoptical/supraorbital **(D)** indicated reduction in *frmpd4* CRISPants (4 dpf). **(E)** Measurement of neuromast distance in the posterior lateral line indicated no significant change after frmpd4 CRISPR or Morpholino knockdown. **(F)** Staining for acetylated tubulin in 4 dpf embryos indicated mild changes to neuronal cells and axonal projections in ventral sensory patches of the otic vesicle and not significant interference with correct neuromast spacing in the posterior lateral line after transient *frmpd4* CRISPR knockdown. Analyzed embryos: 2 wild type controls; 4 *frmpd4* CRISPants.

medRxiv preprint doi: https://doi.org/10.64898/2026.03.27.26349271; this version posted March 30, 2026. The copyright holder for this preprint (which was not certified by peer review) is the author/funder, who has granted medRxiv a license to display the preprint in perpetuity.
It is made available under a CC-BY 4.0 International license .

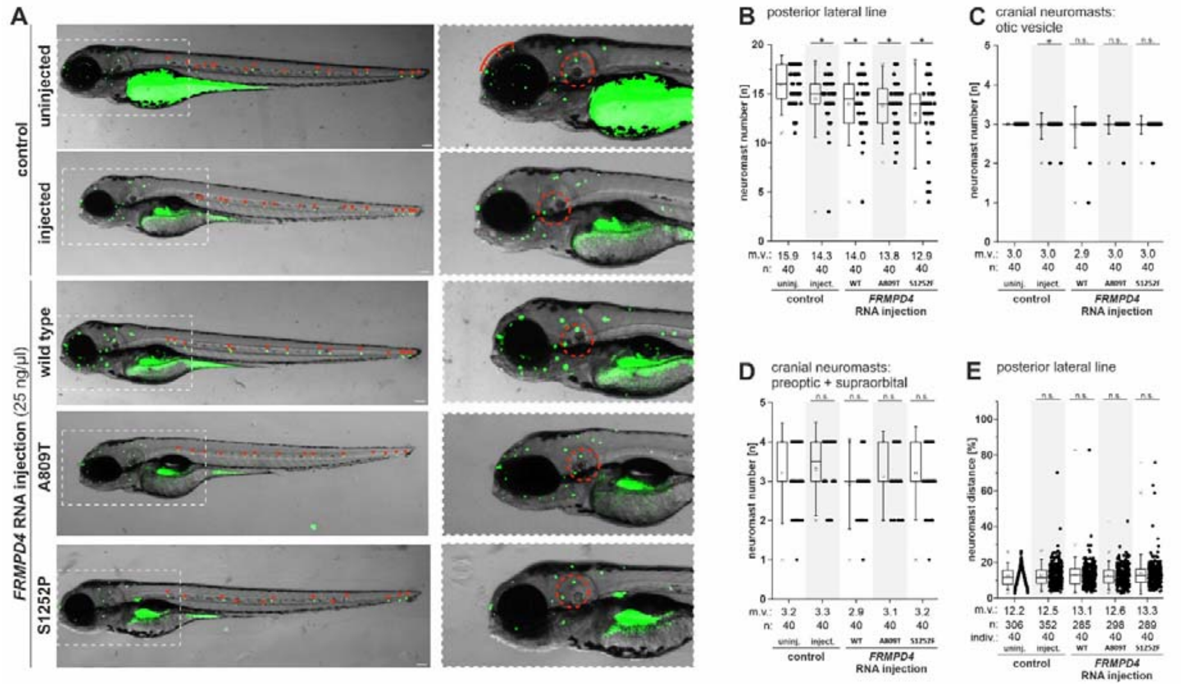

Supplementary Figure 10. *FRMPD4* variant expression in zebrafish via RNA injection.
(A) Brightfield and DASPEI overlay marking hair cells of 4 dpf zebrafish embryos injected with capped mRNAs coding for patient-specific *FRMPD4* variants. Quantification of neuromast number in the posterior lateral line **(B)**, the otic vesicle **(C)** and the preoptical/supraorbital **(D)**, and measurement of neuromast distance in the posterior lateral line **(E)** (Injection concentration: 25ng/ml RNA; injection controls: water instead of RNA). Regions of shown magnifications are outlined with white dashed lines. The otic vesicles are outlined with red dashed lines. DASPEI positive hair cells/ neuromasts of the pLL are marked with red arrowheads. m.v. – mean value; n – amount; indiv. – number of investigated individuals. Statistical significance as calculated by a two-tailed Mann-Whitney U test in comparison to uninjected controls. Statistical significance is depicted as * for U ≤ 0.05, n.s. mark not significant different groups. Scale bars indicate 100µm.

medRxiv preprint doi: https://doi.org/10.64898/2026.03.27.26349271; this version posted March 30, 2026. The copyright holder for this preprint (which was not certified by peer review) is the author/funder, who has granted medRxiv a license to display the preprint in perpetuity.
It is made available under a CC-BY 4.0 International license .

Supplemental references

Adzhubei IA, Schmidt S, Peshkin L, Ramensky VE, Gerasimova A, Bork P, et al. A method and server for predicting damaging missense mutations. Nat Methods. 2010 Apr;7(4):248–9.

Alirezaie N, Kernohan KD, Hartley T, Majewski J, Hocking TD. ClinPred: Prediction Tool to Identify Disease-Relevant Nonsynonymous Single-Nucleotide Variants. Am J Hum Genet. 2018 Oct 4;103(4):474–83.

Andermann P, Ungos J, Raible DW. Neurogenin1 defines zebrafish cranial sensory ganglia precursors. Dev Biol. 2002 Nov 1;251(1):45–58.

Appel B, Korzh V, Glasgow E, Thor S, Edlund T, Dawid IB, et al. Motoneuron fate specification revealed by patterned LIM homeobox gene expression in embryonic zebrafish. Development. 1995 Dec;121(12):4117–25.

Azaiez H, Booth KT, Ephraim SS, Crone B, Black-Ziegelbein EA, Marini RJ, et al. Genomic Landscape and Mutational Signatures of Deafness-Associated Genes. Am J Hum Genet. 2018 Oct 4;103(4):484–97.

Bhandiwad AA, Zeddies DG, Raible DW, Rubel EW, Sisneros JA. Auditory sensitivity of larval zebrafish (Danio rerio) measured using a behavioral prepulse inhibition assay. J Exp Biol. 2013 Sept 15;216(Pt 18):3504–13.

Blader P, Fischer N, Gradwohl G, Guillemot F, Strähle U. The activity of neurogenin1 is controlled by local cues in the zebrafish embryo. Development. 1997 Nov;124(22):4557–69.

Brand AH, Perrimon N. Targeted gene expression as a means of altering cell fates and generating dominant phenotypes. Development. Cambridge, England; 1993 June;118(2):401–15.

Chen S, Francioli LC, Goodrich JK, Collins RL, Kanai M, Wang Q, et al. A genomic mutational constraint map using variation in 76,156 human genomes. Nature. 2024 Jan;625(7993):92–100.

De Sainte Agathe J-M, Filser M, Isidor B, Besnard T, Gueguen P, Perrin A, et al. SpliceAI-visual: a free online tool to improve SpliceAI splicing variant interpretation. Hum Genomics. 2023 Feb 10;17(1):7.

Girotto G, Abdulhadi K, Buniello A, Vozzi D, Licastro D, d'Eustacchio A, et al. Linkage study and exome sequencing identify a BDP1 mutation associated with hereditary hearing loss. PLoS One. 2013;8(12):e80323.

Göpfert MC, Humphris ADL, Albert JT, Robert D, Hendrich O. Power gain exhibited by motile mechanosensory neurons in Drosophila ears. Proc Natl Acad Sci U S A. 2005 Jan 11;102(2):325–30.

Hehlert P, Effertz T, Gu R-X, Nadrowski B, Geurten BRH, Beutner D, et al. NOMPC ion channel hinge forms a gating spring that initiates mechanosensation. Nat Neurosci. 2025 Feb;28(2):259–67.

Honda S, Hayashi S, Imoto I, Toyama J, Okazawa H, Nakagawa E, et al. Copy-number variations on the X chromosome in Japanese patients with mental retardation detected by array-based comparative genomic hybridization analysis. J Hum Genet. 2010 Sept;55(9):590–9.

medRxiv preprint doi: https://doi.org/10.64898/2026.03.27.26349271; this version posted March 30, 2026. The copyright holder for this preprint (which was not certified by peer review) is the author/funder, who has granted medRxiv a license to display the preprint in perpetuity.
It is made available under a CC-BY 4.0 International license .

Hu H, Haas SA, Chelly J, Van Esch H, Raynaud M, de Brouwer APM, et al. X-exome sequencing of 405 unresolved families identifies seven novel intellectual disability genes. Mol Psychiatry. 2016 Jan;21(1):133–48.

Hu J-H, Yang L, Kammermeier PJ, Moore CG, Brakeman PR, Tu J, et al. Preso1 dynamically regulates group I metabotropic glutamate receptors. Nat Neurosci. 2012 June;15(6):836–44.

Ioannidis NM, Rothstein JH, Pejaver V, Middha S, McDonnell SK, Baheti S, et al. REVEL: An Ensemble Method for Predicting the Pathogenicity of Rare Missense Variants. The American Journal of Human Genetics. 2016 Oct;99(4):877–85.

Jao L-E, Wente SR, Chen W. Efficient multiplex biallelic zebrafish genome editing using a CRISPR nuclease system. Proc Natl Acad Sci U S A. 2013 Aug 20;110(34):13904–9.

Karak S, Jacobs JS, Kittelmann M, Spalthoff C, Katana R, Sivan-Loukianova E, et al. Diverse Roles of Axonemal Dyneins in Drosophila Auditory Neuron Function and Mechanical Amplification in Hearing. Sci Rep-Uk. Nature Publishing Group; 2015 Nov 26;5(1):17085.

Landrum MJ, Chitipiralla S, Kaur K, Brown G, Chen C, Hart J, et al. ClinVar: updates to support classifications of both germline and somatic variants. Nucleic Acids Res. 2025 Jan 6;53(D1):D1313–21.

Lauer AM, May BJ. The medial olivocochlear system attenuates the developmental impact of early noise exposure. J Assoc Res Otolaryngol. 2011 June;12(3):329–43.

Lee HW, Choi J, Shin H, Kim K, Yang J, Na M, et al. Preso, a novel PSD-95-interacting FERM and PDZ domain protein that regulates dendritic spine morphogenesis. J Neurosci. 2008 Dec 31;28(53):14546–56.

Li C, Li X, Bi Z, Sugino K, Wang G, Zhu T, et al. Comprehensive transcriptome analysis of cochlear spiral ganglion neurons at multiple ages. Elife [Internet]. 2020 Jan 8;9. Available from: /pmc/articles/PMC7299348/

Li R-K, Li H, Tian M-Q, Li Y, Luo S, Liang X-Y, et al. Investigation of FRMPD4 variants associated with X-linked epilepsy. Seizure. 2024 Mar;116:45–50.

Mazzoli M, Van Camp G, Newton V, Giarbini N, DeClau F, Parving A. Recommendations for the Description of Genetic and Audiological Data for Families with Nonsyndromic Hereditary Hearing Impairment. Audiological Medicine. 2003;1(2):148–50.

McGuire B, Fiorillo B, Ryugo DK, Lauer AM. Auditory nerve synapses persist in ventral cochlear nucleus long after loss of acoustic input in mice with early-onset progressive hearing loss. Brain Res. 2015 Apr 24;1605:22–30.

Orvis J, Gottfried B, Kancherla J, Adkins RS, Song Y, Dror AA, et al. gEAR: Gene Expression Analysis Resource portal for community-driven, multi-omic data exploration. Nat Methods. 2021 Aug;18(8):843–4.

Oza AM, DiStefano MT, Hemphill SE, Cushman BJ, Grant AR, Siegert RK, et al. Expert specification of the ACMG/AMP variant interpretation guidelines for genetic hearing loss. Hum Mutat. 2018 Nov;39(11):1593–613.

Pan H, Zhu F, Chen K, Zhang Y. Genetic analysis of a child with severe intellectual disability caused by a novel variant in the FERM domain of the FRMPD4 protein. J Genet. 2024;103:14.

medRxiv preprint doi: https://doi.org/10.64898/2026.03.27.26349271; this version posted March 30, 2026. The copyright holder for this preprint (which was not certified by peer review) is the author/funder, who has granted medRxiv a license to display the preprint in perpetuity.
It is made available under a CC-BY 4.0 International license .

Petrovski S, Wang Q, Heinzen EL, Allen AS, Goldstein DB. Genic intolerance to functional variation and the interpretation of personal genomes. PLoS Genet. 2013;9(8):e1003709.

Piard J, Hu J-H, Campeau PM, Rzonca S, Van Esch H, Vincent E, et al. FRMPD4 mutations cause X-linked intellectual disability and disrupt dendritic spine morphogenesis. Hum Mol Genet. 2018 Feb 15;27(4):589–600.

Raible DW, Kruse GJ. Organization of the lateral line system in embryonic zebrafish. J Comp Neurol. 2000 May 29;421(2):189–98.

Schrode KM, Dent ML, Lauer AM. Sources of variability in auditory brainstem response thresholds in a mouse model of noise-induced hearing loss. J Acoust Soc Am. 2022 Dec;152(6):3576.

Schrode KM, Muniak MA, Kim Y-H, Lauer AM. Central Compensation in Auditory Brainstem after Damaging Noise Exposure. eNeuro. 2018;5(4):ENEURO.0250-18.2018.

Schubach M, Maass T, Nazaretyan L, Röner S, Kircher M. CADD v1.7: using protein language models, regulatory CNNs and other nucleotide-level scores to improve genome-wide variant predictions. Nucleic Acids Res. 2024 Jan 5;52(D1):D1143–54.

Schwarz JM, Cooper DN, Schuelke M, Seelow D. MutationTaster2: mutation prediction for the deep-sequencing age. Nat Methods. 2014 Apr;11(4):361–2.

Senthilan PR, Piepenbrock D, Ovezmyradov G, Nadrowski B, Bechstedt S, Pauls S, et al. Drosophila auditory organ genes and genetic hearing defects. Cell. 2012 Aug 31;150(5):1042–54.

Sharma Y, Cheung U, Larsen EW, Eberl DF. PPTGAL, a convenient Gal4 P-element vector for testing expression of enhancer fragments in drosophila. Genesis. 2002;34(1–2):115–8.

Shihab HA, Gough J, Cooper DN, Stenson PD, Barker GLA, Edwards KJ, et al. Predicting the functional, molecular, and phenotypic consequences of amino acid substitutions using hidden Markov models. Hum Mutat. 2013 Jan;34(1):57–65.

Shrestha BR, Chia C, Wu L, Kujawa SG, Liberman MC, Goodrich LV. Sensory Neuron Diversity in the Inner Ear Is Shaped by Activity. Cell. 2018 Aug 23;174(5):1229-1246.e17.

Steinhaus R, Proft S, Schuelke M, Cooper DN, Schwarz JM, Seelow D. MutationTaster2021. Nucleic Acids Res. 2021 July 2;49(W1):W446–51.

Takayanagi H, Yuzawa S, Sumimoto H. Structural basis for the recognition of the scaffold protein Frmpd4/Preso1 by the TPR domain of the adaptor protein LGN. Acta Crystallogr F Struct Biol Commun. 2015 Feb;71(Pt 2):175–83.

Taliun D, Harris DN, Kessler MD, Carlson J, Szpiech ZA, Torres R, et al. Sequencing of 53,831 diverse genomes from the NHLBI TOPMed Program. Nature. 2021 Feb;590(7845):290–9.

Tanimoto M, Ota Y, Inoue M, Oda Y. Origin of inner ear hair cells: morphological and functional differentiation from ciliary cells into hair cells in zebrafish inner ear. J Neurosci. 2011 Mar 9;31(10):3784–94.

The All of Us Research Program Genomics Investigators, Manuscript Writing Group, Bick AG, Metcalf GA, Mayo KR, Lichtenstein L, et al. Genomic data in the All of Us Research Program. Nature. 2024 Mar 14;627(8003):340–6.

medRxiv preprint doi: https://doi.org/10.64898/2026.03.27.26349271; this version posted March 30, 2026. The copyright holder for this preprint (which was not certified by peer review) is the author/funder, who has granted medRxiv a license to display the preprint in perpetuity.
It is made available under a CC-BY 4.0 International license .

Thisse C, Thisse B. High-resolution in situ hybridization to whole-mount zebrafish embryos. Nat Protoc. 2008;3(1):59–69.

Trujillano D, Bertoli-Avella AM, Kumar Kandaswamy K, Weiss ME, Köster J, Marais A, et al. Clinical exome sequencing: results from 2819 samples reflecting 1000 families. Eur J Hum Genet. 2017 Feb;25(2):176–82.

Vikhe Patil K, Canlon B, Cederroth CR. High quality RNA extraction of the mammalian cochlea for qRT-PCR and transcriptome analyses. Hear Res. 2015 July;325:42–8.

Vona B, Mazaheri N, Lin S-J, Dunbar LA, Maroofian R, Azaiez H, et al. A biallelic variant in CLRN2 causes non-syndromic hearing loss in humans. Hum Genet. 2021 June;140(6):915–31.

Wang J, Song Q, Yu D, Yang G, Xia L, Su K, et al. Ontogenetic development of the auditory sensory organ in zebrafish (Danio rerio): changes in hearing sensitivity and related morphology. Sci Rep. 2015 Nov 3;5:15943.

Westerfield M. The zebrafish book. A guide for the laboratory use of zebrafish (Danio rerio). University of Oregon; 2000.
